# Impact of manufacturers’ eco-design decisions on the closed-loop supply chain under recycling rate regulations

**DOI:** 10.1371/journal.pone.0314511

**Published:** 2025-02-11

**Authors:** Wenxia Liu, Jiang Jiang, Zhixin Mao, Honglei Liu

**Affiliations:** 1 School of Business, Changshu Institute of Technology, Changshu, Jiangsu, China; 2 School of Management, Fudan University, Shanghai, China; 3 College of Business Administration, Shanghai Business School, Shanghai, China; West Pomeranian University of Technology, POLAND

## Abstract

To address increasingly severe environmental issues, various countries have introduced relevant environmental protection regulations. This paper proposes a new government regulation measure to encourage manufacturers to improve recycling rates. Governments set recycling rate targets and reward-penalty mechanisms. This paper constructs a game model involving a manufacturer and a remanufacturer within a closed-loop supply chain system. It studies the equilibrium decisions in three scenarios: no government intervention, manufacturers not taking improvement measures despite government-set recycling rate targets, and manufacturers adopting ecological design after such targets are established. Results indicate that after governments establish recycling rate target: (1) After manufacturers adopt ecological design, the prices of new and remanufactured products decrease, sales volume increases, and the profits of both manufacturers and remanufacturers rise. Therefore, manufacturers would be well-advised to adopt eco-design strategies to enhance the level of recycling. (2) As the recycling rate target increase, the level of ecological design decreases, and the prices of new and remanufactured products rise. It is recommended that governments initially set lower recycling rate targets and then gradually increase them. (3) With the increase in the reward-penalty coefficient, the level of ecological design rises, and the price of new products first increases and then decreases. When remanufacturing is unrestricted, the prices of remanufactured products decrease; however, when remanufacturing is restricted, the prices of remanufactured products first increase and then decrease. Therefore, governments would be well-advised to establish a relatively high reward-penalty coefficient.

## 1 Introduction

With the advance of science and technology and the improvement of living standards, electrical and electronic products and automobiles have become an indispensable part of a modern, materialist, consumer-oriented society. However, the sharp increase in the number of Waste Electrical and Electronic Equipment (WEEE) and end-of-life vehicles pose a serious threat to the environment. To address the increasingly severe environmental issues, several countries have introduced relevant environmental regulations and ecological standards to guide companies to adopt proactive eco-design measures and improve the recycling rate of their products.

Governments are developing measures to promote eco-design and circular economy. In China, the “Implementation Plan for the Extended Producer Responsibility System” proposes that by 2025, the relevant laws and regulations for this manufacturer responsibility system will be basically complete. If implemented as intended, the widespread adoption of eco-design will be achieved, and the average recycling rate of waste products will reach 50%. In Europe, over six million vehicles reach the end of their life each year. To address this, the European Commission released “End-of-Life Vehicles” regulations in July 2023 that specify requirements for reusability and recyclability in vehicle manufacture. Specifically, that the proportion of recycled materials to be used in vehicle design and production, including the overall reusability rate of vehicles will be no less than 85%. In March 2024, China implemented the “Road Vehicles—Requirements and Calculation Methods for Reusability and Recyclability” (GB/T 19515–2023), which stipulates that the reusability rate of vehicles should be no less than 85%.

In response to government regulations on recycling rates, companies can adopt designs that facilitate disassembly [1] and use recycled materials during the design stage [2], known as eco-design. In February 2023, Intel announced the launch of green commercial computers in collaboration with partners such as Tsinghua Tongfang and Acer, adopting eco-design to improve product recyclability. In September of the same year, Audi showcased the incorporation of recycling concepts into the design phase of automobiles at the “Sustainable Design Summit”. These policies and practices show that recycling rate and ecological design have become the focus of governments and enterprises.

In the era of the circular economy, government regulations and eco-design have garnered increasing attention from academia. Extended Producer Responsibility (EPR) plays a crucial role as a policy tool in improving product recycling rates. Recent studies have demonstrated a strong positive correlation between EPR policies and recycling rates. Tumu et al. [3] conducted a global study revealing that countries implementing EPR regulations typically achieve higher recycling rates. This finding is corroborated by Rubio et al. [4], who observed positive impacts of EPR policies on increasing recycling rates in packaging waste management in Portugal and Spain. The impact of EPR policies on recycling rates is evident across various product categories. Shimada & Van Wassenhove [5] showed that the recycling efficiency in Japan’s home appliance industry significantly improved following the implementation of EPR programs. Similarly, Hou et al. [6], in evaluating a new EPR mode for electronic waste in China, found that mandatory recycling modes can achieve higher recycling rates. Colelli et al. [7] analyzed the cost efficiency and recycling effectiveness of EPR schemes in the European packaging waste system, finding that non-competitive systems were more successful in improving recycling rates. Son et al. [8] examined a closed-loop supply chain planning model for rare metals in South Korea, finding that government storage and EPR recycling obligations significantly increased the amount of recycled flow and reduced manufacturing costs. Turner and Nugent [9] analyzed EPR policies for single-use batteries in the EU, Canada, and the USA, highlighting the limitations of these policies in enhancing recycling rates. However, the impact of EPR policies on recycling rates may vary depending on implementation methods and market conditions. Zan & Zhang [10] demonstrated that different recycling channel structures lead to variations in recycling rates. Kuo et al. [11] emphasized the importance of recycling funds, finding that increasing these funds can improve recycling rates. This indicates that the specific design of EPR policies is crucial to their effectiveness. Notably, while EPR policies generally improve recycling rates, they may have complex effects on other aspects. Cao et al. [12] found that although EPR helps increase collection rates, overly stringent collection targets might inhibit remanufacturing activities. This suggests the need for a balanced approach when formulating EPR policies. These studies provide an important background for understanding the impact of EPR policies on closed-loop supply chain firms’ decisions. However, further research is needed to explore how other government regulatory measures influence the decision-making of closed-loop supply chain members.

End-of-Life Vehicle (ELV) recycling has become a crucial issue for the sustainable development of the global automotive industry. Recent studies indicate significant variations in ELV recycling policies and practices across different countries and regions, with a general trend towards improving recycling rates and promoting resource circularity. Yi & Lee [13] revealed the economic challenges of ELV recycling policies in Korea, emphasizing the importance of the dismantling stage in the recycling process and recommending targeted financial support to improve overall recycling rates. This finding resonates with Sulaiman et al.’s study in Malaysia [14], which found that Authorized Automotive Treatment Facilities (AATF) can achieve recycling rates of up to 90%, highlighting the key role of specialized processing facilities in enhancing ELV recycling efficiency. Bhari et al. [15] compared ELV material flows between Japan and the EU, revealing differences in recycling and energy recycling rates despite both regions having established comprehensive ELV management systems. These disparities likely stem from differences in policy orientations and technological approaches, suggesting the need for a deeper understanding of regional ELV recycling practices. Soo et al. [16] extended this cross-national perspective by analyzing the impact of ELV regulations on vehicle material circularity in Europe, Japan, Australia, and the US. Their study emphasized the limitations of current recycling rate-oriented policies in promoting genuine circular economy principles.

Scholars have studied the impact of various regulatory measures on recycling and remanufacturing, such as the WEEE (Waste Electrical and Electronic Equipment) directive, taxes and processing fees, and subsidies. Wang et al. [17] examined the effects of government intervention measures on waste electrical and electronic equipment (WEEE) recycling in China, concluding that strict supervision and severe penalties are more effective in promoting WEEE recycling than mere incentives. This finding underscores the importance of policy enforcement, not just policy formulation. Plambeck & Wang [18] compared different types of e-waste regulations, noting that “fee-upon-disposal” regulations (such as individual extended producer responsibility) are more effective in motivating manufacturers to eco-design. However, they may fail to reduce the frequency of new product introductions in highly competitive product categories, highlighting the need for policy design to consider market complexities. Chen & Sheu [19] further proposed a strategy of gradually raising environmental standards to encourage manufacturers to progressively improve product recyclability, providing a long-term perspective for policy-making. Shittu et al. [20] emphasized the leading role of the EU WEEE Directive in driving high recycling targets, while also pointing out that incomplete regulatory coverage and weak enforcement are major challenges in global WEEE management. This suggests that successful policies require comprehensive regulatory frameworks and effective implementation mechanisms. Zhang et al. [21] and Zhang & Zhang [22] focused on the impact of government subsidies on remanufacturing supply chains. They found that government subsidies can effectively promote remanufacturing, but their effects are influenced by market demand fluctuations and supply chain cooperative behaviors. Notably, Zhang et al. [21] indicated that in the early stages of the remanufacturing industry, governments may need to impose high taxes on manufacturers to accumulate remanufacturing subsidies, while establishing a levy-subsidy mechanism becomes crucial for maintaining sustainable industry development as the sector matures and consumer environmental awareness increases. Arslan et al. [23] revealed an association between government ideology and e-waste recycling rates, finding that left-wing or center-wing party governance correlates with higher recycling rates. This insight provides a new perspective on understanding the potential political drivers of policy-making, emphasizing that policymakers’ political inclinations may influence the effectiveness of environmental policies. These studies provide valuable insights into understanding the impact of government regulations on closed-loop supply chain firms’ decisions. However, further research is needed to explore how government regulatory measures influence manufacturers’ eco-design decisions.

Scholars have put forward some important viewpoints regarding the relationship between government regulations and eco-design. Studies by Compagnoni [24] and Joltreau [25] indicate that while Extended Producer Responsibility (EPR) has achieved significant results in downstream objectives such as waste management, its effectiveness in promoting eco-design remains limited. Huang et al. [26] reveal that EPR’s impact on product design is multifaceted, potentially involving trade-offs between recyclability and durability, which may lead to unexpected environmental consequences. Researchers have proposed various explanations and solutions to address these issues. Compagnoni [24] attributes the limited effectiveness to insufficient allocation of individual responsibility to producers, while Gui et al. [27] find that collective EPR implementation may be more effective than individual implementation in promoting eco-design. To address these challenges, some countries have begun experimenting with new policy tools. For instance, Micheaux and Aggeri [28] studied the eco-modulation mechanism introduced in the French WEEE industry, finding that although its direct impact on product design is not obvious, it has produced important indirect effects. However, Lifset et al. [29] point out that restoring eco-design incentives in EPR through eco-modulation still faces challenges. Zheng et al. [2] propose a new incentive scheme design model aimed at simultaneously achieving recycling cost efficiency and eco-design incentives, with a particular focus on EPR implementation for waste tires. Research by Pruess [30] further emphasizes the complexity and diversity of EPR policy design, indicating the need for more comprehensive management and implementation strategies to effectively promote eco-design. These studies reveal the complexity of the impact mechanism of government regulations on eco-design, providing an important theoretical foundation for our research. However, further exploration is needed to understand how manufacturers make eco-design decisions under specific recycling rate regulations.

Scholars’ research provides important insights into the relationship between government subsidies, eco-design, and recycling rates. Both Yu et al. [31] and Xiao et al. [32] emphasize the important role of government subsidy policies in improving recycling rates and promoting eco-design. Yu et al. [31] found that fixed-cost subsidies for recyclers generally lead to higher recycling rates and social welfare, while also noting that eco-design can effectively increase waste recycling rates. Xiao et al. [32] further pointed out that recycling subsidies have positive effects on both eco-design and recycling, while the effectiveness of eco-design subsidies depends on whether manufacturers develop recycling-oriented eco-designs. These findings highlight the complexity and importance of policy incentives in promoting eco-design and improving recycling rates. Liao & Chuang’s [33] research explored the innovation of eco-friendly electronic products from a product design perspective, noting that sustainability attributes, including recyclability, have a significant impact on consumer choices. This suggests that eco-design not only improves the environmental performance of products but also meets consumer demands, potentially increasing product recycling rates. The eco-efficiency assessment method proposed by Chin et al. [34] provides policymakers with a powerful tool to formulate more effective policies by comparing the eco-efficiency of different waste treatment scenarios (including different recycling rates). These studies provide an important basis for understanding the relationship between policy incentives, eco-design, and recycling rates. However, further research is needed to explore how manufacturers weigh different factors to make eco-design decisions under government recycling rate regulations, and how these decisions affect the operation of the closed-loop supply chain.

In summary, existing research provides rich insights into the relationship between government regulations, eco-design, and recycling rates. However, there are still some important research gaps. In particular, manufacturers’ decisions under government recycling rate regulations, and how these decisions affect the operation of the closed-loop supply chain, have not been fully studied. Therefore, this paper aims to explore the following research questions: (1) Manufacturers’ decisions under government recycling rate regulations. (2) The impact of manufacturers’ eco-design decisions on the operation of the closed-loop supply chain. (3) The differences in equilibrium decisions of closed-loop supply chain members when remanufacturing is unrestricted and when it is restricted. The innovations of this paper are outlined as follows:

(1) To address increasingly severe environmental issues, we propose a new government regulation measure to encourage manufacturers to improve recycling rates. Governments set targets for the recycling rate of new products, imposing penalties on products that fall below the target and providing rewards for those that exceed it. The recycling rate target are set above the current levels of recycling achieved by the enterprises.
(2) In response to the recycling rate targets set by governments, this paper innovatively proposes that companies may adopt two measures. (i) Manufacturers take no action and accept prescribed penalties. (ii) Manufacturers adopt eco-design strategies to improve the recycling rate. After manufacturers adopt eco-design strategies, if the product’s recycling rate exceeds the target, the company receives rewards; if the product’s recycling rate falls below the target, the company incurs penalties.
(3) This paper examines the impact of recycling rate target and reward-penalty coefficient on companies’ equilibrium decisions, comparing these decisions across three scenarios: before government regulation, after regulation with manufacturers not implementing improvement measures, and after regulation with manufacturers adopting eco-design strategies. The findings provide valuable insights for government in setting recycling rate target and structuring reward-penalty coefficient.

The remainder of this paper is structured as follows: Section 2 describes the problem and outlines the basic assumptions. Section 3 establishes game models for three scenarios: before government regulation of recycling rates, after regulation when manufacturers do not implement improvement measures, and after regulation when manufacturers adopt eco-design strategies. Section 4 analyzes the impact of recycling rate target and reward-penalty coefficient on equilibrium decisions, comparing these decisions across the three scenarios. Section 5 conducts a simulation analysis to examine the effects of recycling rate target and reward-penalty coefficient on equilibrium decisions, comparing the outcomes across the three states. Finally, Section 6 presents the conclusions and implications, summarizing the research findings and proposing recommendations based on the results.

## 2 Problem description and basic assumptions

### 2.1 Problem description

This paper constructs a closed-loop supply chain system consisting of a manufacturer and a remanufacturer. The manufacturer produces new products, and the remanufacturer recycles end-of-life products and produces remanufactured products. Information is completely symmetrical between manufacturers and remanufacturers, both of whom aim to maximize profits in their decision-making. The manufacturer acts as the leader in the closed-loop supply chain, while the remanufacturer is the follower, engaging in a Stackelberg game. Both manufacturers and remanufacturers are risk-neutral and fully rational [35, 36].

The market with which we are concerned involves the heterogeneous demand for both new and remanufactured products. New products and remanufactured products are functionally interchangeable, assuming that each consumer can purchase at most one unit of either a new product or a remanufactured product [37]. Due to differences in cost, quality, and branding between new and remanufactured products, many consumers perceive remanufactured products as low-quality refurbished items and are reluctant to purchase them [38, 39]. Empirical analyses by Guide and Li [40] and Yao Chen [41] have shown that consumers’ willingness to pay for remanufactured products is lower than for new products. Therefore, this paper assumes that consumers’ willingness to pay for remanufactured products is lower than for new products. The consumers’ willingness to pay for the new product is *θ*. According to the assumption by Ferrer and Swaminathan [42], *θ* follows a uniform distribution over the interval [0, 1]. Consumers value new and remanufactured products differently and thus seek to pay differential rates. The price of new products is denoted by *p*_*n*_, and the production cost is *c*_*n*_. To ensure product profitability, the price of new products must be less than the maximum consumer valuation, *c*_*n*_ < *p*_*n*_ < 1. Consumers value remanufactured products lower than new products, with a discount factor *α*, 0 < *α* < 1, where *αθ* represents the consumer valuation of remanufactured products [43]. The price of remanufactured products is *p*_*r*_, and the production cost is *c*_*r*_. The production cost and price of remanufactured products are lower than those of new products, *c*_*r*_ < *c*_*n*_, *p*_*r*_ < *p*_*n*_. The cost savings from remanufacturing are represented by *s*_*r*_,*s*_*r*_ = *c*_*n*_ − *c*_*r*_. The utility for consumers purchasing new products is given by *U*_*n*_ = *θ* − *p*_*n*_, and the utility for purchasing remanufactured products is *U*_*r*_ = *αθ* − *p*_*r*_. Consumers’ choice between new products and remanufactured products depends on the value utility of these two types of products.

The sales prices of new and remanufactured products will determine consumers’ purchasing behavior. Different product prices result in various forms of product demand. There may be two distinct forms of product demand in the consumer market: (1) demand only for new products, and (2) demand for both new and remanufactured products. Scenario (1) indicates that the manufacturer sells new products. Scenario (2) indicates that the manufacturer sells new products while the remanufacturer recycles end-of-life products and sells remanufactured products. This study explores differential pricing strategies within a closed-loop supply chain system based on demand functions under different market scenarios.

(1) When *U*_*n*_ > 0 and *U*_*n*_ > *U*_*r*_, meaning $\text{max}\{p_{n},\frac{p_{n}-p_{r}}{1-\alpha }\}<\theta <1$, the consumer purchases the new product.
(2) When *U*_*r*_ > 0 and *U*_*r*_ > *U*_*n*_, meaning $\frac{p_{r}}{\alpha }<\theta <\frac{p_{n}-p_{r}}{1-\alpha }$, the consumer purchases the remanufactured product.

By analyzing consumer choice behavior, demand functions under different market scenarios are derived.

In the case of choice behavior (1), when $\frac{p_{n}-p_{r}}{1-\alpha }<p_{n}<\theta <1$ is satisfied, we obtain $\alpha <\frac{p_{r}}{p_{n}}$, and at this point, $\left\{\theta |\frac{p_{r}}{\alpha }<\theta <\frac{p_{n}-p_{r}}{1-\alpha }\right\}$ is an empty set, meaning that consumers do not purchase remanufactured products. Only new product demand exists in the market. This leads to Lemma 1.

Lemma 1: When $\alpha <\frac{p_{r}}{p_{n}}$, only new product demand exists in the market, with its demand function as follows:

$$q_{n}=\int \begin{array}{c} 1\\ p_{n} \end{array}d\theta =1-p_{n}$$

In the case of choice behavior (1), when $p_{n}<\frac{p_{n}-p_{r}}{1-\alpha }<\theta <1$ is satisfied, we derive $\frac{p_{r}}{p_{n}}<\alpha <1-\frac{p_{n}-p_{r}}{\theta }$, and at this point, $\left\{\theta |\frac{p_{r}}{\alpha }<\theta <\frac{p_{n}-p_{r}}{1-\alpha }\right\}$ is a non-empty set, indicating that consumers purchase remanufactured products. There is demand for both new and remanufactured products in the market. This leads to Lemma 2.

Lemma 2: When $\frac{p_{r}}{p_{n}}<\alpha <1-\frac{p_{n}-p_{r}}{\theta }$, there is demand for both new and remanufactured products in the market. The demand functions for the two products are as follows: The demand function for new products is:

$$q_{n}=\int \begin{array}{c} 1\\ \frac{p_{n}-p_{r}}{1-\alpha } \end{array}d\theta =1-\frac{p_{n}-p_{r}}{1-\alpha }$$

The demand function for remanufactured products is:

$$q_{r}=\int \begin{array}{c} \frac{p_{n}-p_{r}}{1-\alpha }\\ \frac{p_{r}}{\alpha } \end{array}d\theta =\frac{\alpha p_{n}-p_{r}}{\alpha (1-\alpha )}$$

To enhance the level of product recycling, government has established recycling rate target that exceed the current level of recycling achieved by enterprises. As the level of product recycling increases, government will raise these targets. For instance, the European Union’s End-of-Life Vehicles Directive sets a recycling rate target for vehicles: starting from January 1, 2006, at least 85% of the average weight of each scrapped vehicle must be reusable annually. This target was increased to 95% starting from January 1, 2015. Let *m*_*p*_ represent the current level of product recycling rate, *m*_*o*_ denote government-mandated recycling rate target. The recycling rate target set by government is higher than the current level of recycling achieved by enterprises, *m*_*o*_ > *m*_*p*_. *m* represents the task of increasing the recycling rate, *m* = *m*_*o*_ − *m*_*p*_ > 0.

What policies can promote manufacturers in achieving the targets set by government? Research on previous policies shows that the Chinese government has imposed a processing fund on producers to encourage the recycling and treatment of waste electrical and electronic products, and provided subsidies to treatment enterprises. For example, in May 2012, the Chinese government issued the “Management Measures for the Collection and Use of the Processing Fund for Waste Electrical and Electronic Products” which imposed a processing fund on producers of electrical and electronic products and provided subsidies to treatment enterprises. In 2024, the Chinese government allocated 7.5 billion yuan to continue supporting the recycling and treatment of waste electrical and electronic products through a “reward instead of subsidy” approach. Therefore, this paper suggests that government set recycling rate target to enhance the recycling of products. For products exceeding the recycling rate target, government offers rewards, while for those falling below the target, penalties are imposed.

Governments set recycling rate target and reward-penalty coefficient, providing rewards for products that exceed the recycling rate target and imposing penalties on those that fall below the target. *δ*_*n*_ represents the reward-penalty coefficient, *δ*_*n*_ > 0. The recycling rate target set by government is higher than the current level of recycling achieved by enterprises, *m*_*o*_ − *m*_*p*_ = *m* > 0. If manufacturers do not take improvement measures, they will be required to pay a fine. The penalty amount is *mδ*_*n*_.

Manufacturers can adopt eco-design to improve the recycling rate of their products. Eco-design involves using materials that are easy to recycle and implementing designs that facilitate assembly and disassembly when producing new products. Eco-design can enhance the recycling rate of end-of-life products and reduce the production costs of remanufactured products. Implementing eco-design requires manufacturers to invest a certain amount of initial capital. Let *τ* denote the eco-design level of new products, 0 < *τ* < 1. *k* represents the difficulty coefficient of eco-design, and implementing eco-design requires an initial investment. According to the literature by Kim and Chhajed [44], the initial investment for eco-design is *kτ*^2^/2. The square term *τ*^2^ indicates that the required investment grows at an increasing rate as the level of eco-design improves. *δ*_*r*_ is the cost variation coefficient of remanufactured products, reflecting the impact of eco-design on the costs of remanufactured products, *δ*_*r*_ > 0. After adopting eco-design, the production cost of remanufactured products is *c*_*n*_ − *s*_*r*_ − *δ*_*r*_*τ*, while the production cost of new products is *c*_*n*_ − *δ*_*n*_(*τ* − *m*). *δ*_*n*_ represents reward-penalty coefficient, and government’s reward-penalty mechanism aligns with expression (*τ* − *m*)*δ*_*n*_ [45, 46]. *m* denotes the task of increasing the recycling rate. *τ* > *m* indicates that the increase in recycling rate resulting from eco-design exceeds the task of increasing the recycling rate, meaning that the product’s recycling rate is above the target value, and government rewards the manufacturer. *τ* < *m* indicates that the increase in recycling rate resulting from eco-design is less than the task of increasing the recycling rate, meaning that the product’s recycling rate is below the target value, and the manufacturer is required to pay a fine.

Considering that remanufacturing may be limited by the quantity of recovered end-of-life products, this paper examines two scenarios: remanufacturing without restrictions and remanufacturing with restrictions. γ* represents the ratio of the number of remanufactured products to the number of new products. *γ* represents the ratio of the number of products available for remanufacturing to the number of new products. *γ* ≥ γ* indicates that remanufacturing is unrestricted. *γ* < γ* indicates that remanufacturing is restricted. For simplicity in calculations, we assume that $\bar{\alpha }\equiv 1-\alpha$, $\bar{\gamma }\equiv 1-\gamma$, ${\bar{c}}_{n}\equiv 1-c_{n}$. The descriptions of parameters and decision variables are shown in Table 1.

**Table 1:** Parameters and decision variables.

Notation	Definition
*n*, *r*:	Subscript Subscript *n* represents new product, *r* represents remanufactured product
$p_{n}^{i}\left(\forall i\right)$ , $p_{r}^{i}\left(\forall i\right)$:	Selling prices of new product and remanufactured product
*c*_*n*_, *c*_*r*_:	The production cost of new product, the production cost of remanufactured product
*s*_*r*_:	The cost saving from remanufactured product, *s*_*r*_ = *c*_*n*_- *c*_*r*_
*α*:	The value discount factor of remanufactured products relative to new products from the consumers’ perspective. 0 < *α* < 1
*m*_*p*_:	The current level of product reuse rate
*m*_*o*_:	The government-mandated reuse rate target
*m*:	The task of increasing reuse rate, *m*_*o*_ − *m*_*p*_ = *m* > 0
*τ*:	The eco-design level of new product, 0 < *τ* < 1
*k*:	Eco-design difficulty coefficient.
*δ*_*n*_:	The government’s reward-penalty coefficient, *δ*_*n*_ > 0
*δ*_*r*_:	The cost variation coefficient of remanufactured products, *δ*_*r*_ > 0
*γ*^i^*(∀*i*):	Ratio of the number of remanufactured products to the number of new products
*γ*:	Ratio of the number of products available for remanufacturing to the number of new products.
$q_{n}^{i}\left(\forall i\right)$ , $q_{r}^{i}\left(\forall i\right)$	The sales volumes of new product, The sales volumes of remanufactured product
$\pi_{M}^{i}\left(\forall i\right)$ , $\pi_{R}^{i}\left(\forall i\right)$, *π*^*i*^(∀*i*):	Profits of the manufacturer, remanufacturer, and the closed-loop supply chain

### 2.2 Basic assumptions

Assumption 1: The information is completely symmetric between manufacturers and remanufacturers, both of whom are risk-neutral and fully rational, making optimal decisions based on maximizing expected profits [35]. The manufacturer acts as the leader in the closed-loop supply chain, while the remanufacturer is the follower, and both engage in a Stackelberg game.

Assumption 2: It is assumed that each consumer in the market will purchase at most one unit of either a new product or a remanufactured product, with new products and remanufactured products being functionally substitutable [37].

Assumption 3: Considering the differences in cost, quality, and branding between new products and remanufactured products, many consumers perceive remanufactured products as low-quality refurbished items and are reluctant to purchase them. Therefore, it is assumed that consumers’ willingness to pay for remanufactured products is lower than their willingness to pay for new products [38–41].

Assumption 4: Government provides incentives for products that exceed the recycling rate target and imposes penalties for products that fall below the target. The reward-penalty mechanism aligns with the expression (*τ* − *m*)*δ*_*n*_, where *δ*_*n*_ represents the reward-penalty coefficient. *τ* > *m* indicates that the product’s recycling rate is above the target value, and government grants rewards to manufacturers. Conversely, *τ* < *m* indicates that the product’s recycling rate is below the target value, resulting in penalties for the manufacturer [45, 46].

## 3 Game model

**Fig 1:**
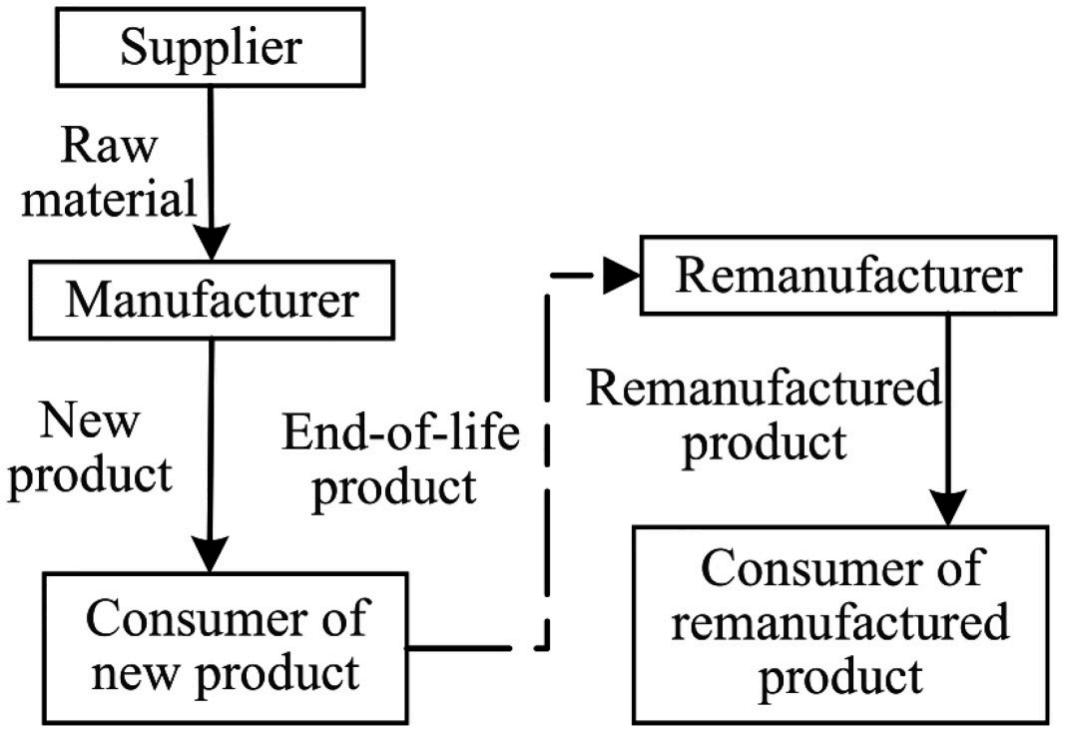
Structural framework diagrams of N mode.

**Fig 2:**
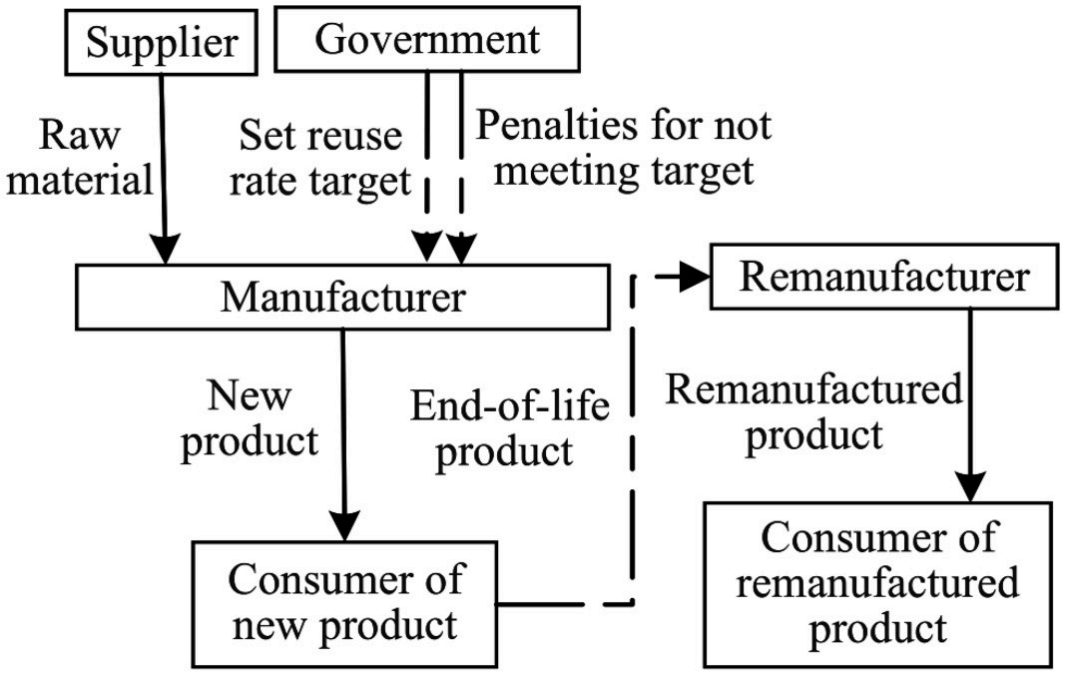
Structural framework diagrams of P mode.

**Fig 3:**
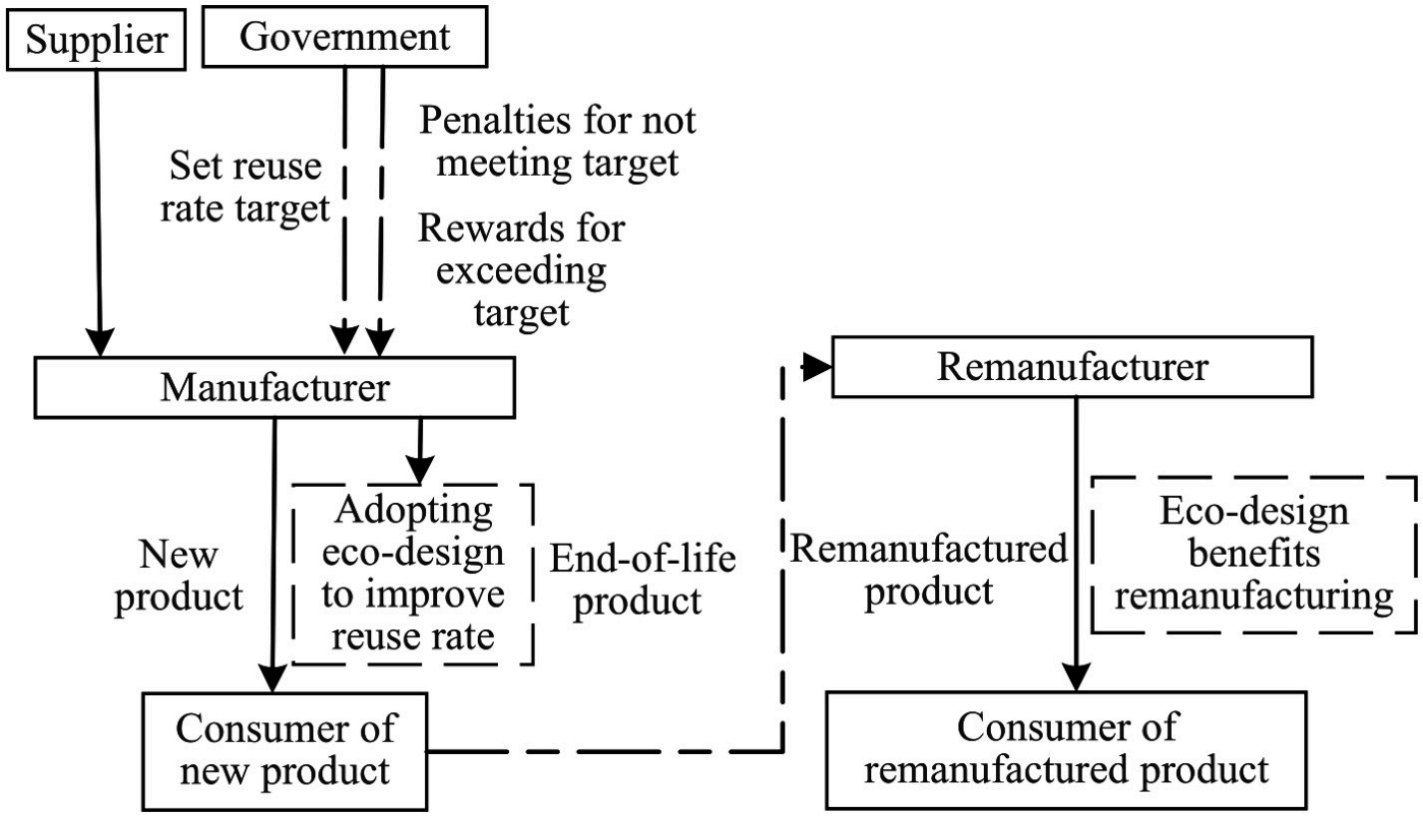
Structural framework diagrams of E mode.

In the closed-loop supply chain system consisting of manufacturers and remanufacturers, manufacturers produce new products while remanufacturers produce remanufactured products. Both new and remanufactured products are sold simultaneously in the market. Government mandates a recycling rate target for new products that is higher than the current recycling rate. Manufacturers have the option to either forgo improvements and pay fines or adopt eco-design measures to improve the recycling rate of their products. This study investigates the interactions between manufacturers and remanufacturers under three different scenarios: (1) the N mode, representing the game between manufacturers and remanufacturers before government sets the recycling rate target. The structural framework diagrams of N mode is shown in Fig 1. (2) the P mode, where government sets the recycling rate target, and manufacturers do not take improvement measures and accept economic penalties. The structural framework diagrams of P mode is shown in Fig 2. (3) the E mode, where government sets the recycling rate target, and manufacturers adopt eco-design to improve the recycling rate of their products. The structural framework diagrams of E mode is shown in Fig 3. Manufacturers decide the price of new products, and remanufacturers decide the price of remanufactured products. Considering the uncertainty of the quantity of recovered products, the analysis is conducted under two conditions: unrestricted remanufacturing and restricted remanufacturing.

### 3.1 N mode game model

In the N mode, the government has not yet set a recycling rate target for new products. The profit maximization function for the manufacturer is:

$$\begin{array}{c} \begin{array}{c} \text{max}\;\pi_{M}^{N}=q_{n}(p_{n}-c_{n}) \end{array} \end{array}$$ (1)

The profit maximization function of the remanufacturer is:

$$\begin{array}{c} \begin{array}{c} \text{max}\;\pi_{R}^{N}=q_{r}(p_{r}-c_{n}+s_{r})\\ s.t.q_{r}\leq \gamma q_{n} \end{array} \end{array}$$ (2)

Construct the Lagrange function:

$$\begin{array}{c} \begin{array}{c} L\left(p_{r},\lambda \right)=\pi_{R}^{N}+\lambda \left(\gamma q_{n}-q_{r}\right) \end{array} \end{array}$$ (3)

λ is the Lagrange multiplier, and λ ≥ 0.

The manufacturer decides the selling price of the new product *p*_*n*_, and the remanufacturer decides the selling price of the remanufactured product *p*_*r*_. Eq (1) is a concave function with respect to *p*_*n*_, while Eqs (2) and (3) are concave functions with respect to *p*_*r*_.

The manufacturer sets the sales price *p*_*n*_ for new products, and the remanufacturer sets the sales price *p*_*r*_ for remanufactured products. Eq (1) is a concave function with respect to *p*_*n*_, and Eqs (2) and (3) are concave functions with respect to *p*_*r*_. To achieve profit maximization, the following conditions must be met: $\frac{\partial^{2}\pi_{M}^{N}}{\partial {p_{n}}^{2}}=-\frac{2}{\bar{\alpha }}<0$, $\frac{\partial^{2}\pi_{R}^{N}}{\partial {p_{r}}^{2}}=-\frac{2}{\alpha \bar{\alpha }}<0$, $\frac{\partial^{2}L\left(p_{r},\lambda \right)}{\partial {p_{r}}^{2}}=-\frac{2}{\alpha \bar{\alpha }}<0$.

Taking the first-order partial derivative of Eq (1) with respect to *p*_*n*_, we obtain:

$$\begin{array}{c} \begin{array}{c} \frac{\partial \pi_{M}^{N}}{\partial p_{n}}=1-\frac{p_{n}-c_{n}}{\bar{\alpha }}-\frac{p_{n}-p_{r}}{\bar{\alpha }} \end{array} \end{array}$$ (4)

1) When *γ* ≥ *γ*^N*^, λ = 0, indicating that remanufacturing is unrestricted.

Taking the first-order partial derivative of Eq (2) with respect to *p*_*r*_, we obtain:

$$\begin{array}{c} \begin{array}{c} \frac{\partial \pi_{R}^{N}}{\partial p_{r}}=\frac{\alpha p_{n}-p_{r}}{\bar{\alpha }\alpha }-\frac{p_{r}-c_{n}+s_{r}}{\bar{\alpha }\alpha } \end{array} \end{array}$$ (5)

By setting Eqs (4) and (5) to zero and solving simultaneously, the optimal solution is obtained:

The selling price of new products is $p_{n}^{N*}=\frac{2\bar{\alpha }+3c_{n}-s_{r}}{3+\bar{\alpha }}$, the selling price of remanufactured products is $p_{r}^{N*}=\frac{\alpha \bar{\alpha }+(2+\alpha )c_{n}-2s_{r}}{3+\bar{\alpha }}$, the sales volume of new products is $q_{n}^{N*}=\frac{\bar{\alpha }+\bar{\alpha }{\bar{c}}_{n}-s_{r}}{3\bar{\alpha }+{\bar{\alpha }}^{2}}$, the sales volume of remanufactured products is $q_{r}^{N*}=\frac{s_{r}+\bar{\alpha }(\alpha -2c_{n}+s_{r})}{\alpha \bar{\alpha }(3+\bar{\alpha })}$, the profit of the manufacturer is $\pi_{M}^{N*}=\frac{{\left(\bar{\alpha }(1+{\bar{c}}_{n})-s_{r}\right)}^{2}}{\bar{\alpha }{\left(3+\bar{\alpha }\right)}^{2}}$, the profit of the remanufacturer is $\pi_{R}^{N*}=\frac{{\left(s_{r}+\bar{\alpha }(\alpha -2c_{n}+s_{r})\right)}^{2}}{\alpha \bar{\alpha }{\left(3+\bar{\alpha }\right)}^{2}}$, the total profit of the closed-loop supply chain is $\pi^{N*}=\frac{\alpha {\left(\bar{\alpha }(1+{\bar{c}}_{n})-s_{r}\right)}^{2}+{\left(s_{r}+\bar{\alpha }(\alpha -2c_{n}+s_{r})\right)}^{2}}{\alpha \bar{\alpha }{\left(3+\bar{\alpha }\right)}^{2}}$, $\gamma^{N*}=\frac{q_{r}^{N*}}{q_{n}^{N*}}=\frac{s_{r}+\bar{\alpha }(\alpha -2c_{n}+s_{r})}{\alpha (\bar{\alpha }(2-c_{n})-s_{r})}$.

2) When *γ* < *γ*^N*^, λ ≠ 0, indicating that remanufacturing is restricted.

Taking the first-order partial derivatives of Eq (3) with respect to λ and *p*_*r*_, we obtain:

$$\begin{array}{c} \begin{array}{c} \frac{\partial L(p_{r},\lambda )}{\partial \lambda }=\gamma (1-\frac{p_{n}-p_{r}}{\bar{\alpha }})-\frac{\alpha p_{n}-p_{r}}{\bar{\alpha }\alpha } \end{array} \end{array}$$ (6)

$$\begin{array}{c} \begin{array}{c} \frac{\partial L(p_{r},\lambda )}{\partial p_{r}}=\frac{\lambda +\alpha \gamma \lambda +c_{n}+\alpha p_{n}-2p_{r}-s_{r}}{\alpha -\alpha^{2}} \end{array} \end{array}$$ (7)

By setting Eqs (4), (6) and (7) to zero and solving simultaneously, the optimal solution is obtained:

The selling price of new products is $p_{n}^{N•}=\frac{\bar{\alpha }+(1+\alpha \gamma )c_{n}}{2-\alpha \bar{\gamma }}$, the selling price of remanufactured products is $p_{r}^{N•}=\frac{\alpha (\bar{\alpha }\bar{\gamma }+(1+\gamma )c_{n})}{2-\alpha \bar{\gamma }}$, the sales volume of new products is $q_{n}^{N•}=\frac{{\bar{c}}_{n}}{2-\alpha \bar{\gamma }}$, the sales volume of remanufactured products is $q_{r}^{N•}=\frac{\gamma {\bar{c}}_{n}}{2-\alpha \bar{\gamma }}$, the profit of the manufacturer is $\pi_{M}^{N•}=\frac{\bar{\alpha }{\bar{c}}_{n}^{2}}{{\left(2-\alpha \bar{\gamma }\right)}^{2}}$, the profit of the remanufacturer is $\pi_{R}^{N•}=\frac{\gamma {\bar{c}}_{n}(\bar{\alpha }(\alpha \bar{\gamma }-2c_{n})+(2-\alpha \bar{\gamma })s_{r})}{{\left(2-\alpha \bar{\gamma }\right)}^{2}}$, the total profit of the closed-loop supply chain is $\pi^{N•}=\frac{{\bar{c}}_{n}(\bar{\alpha }\left(\alpha \gamma \bar{\gamma }+{\bar{c}}_{n}-2\gamma c_{n}\right)+\gamma \left(2-\alpha \bar{\gamma }\right)s_{r})}{{\left(2-\alpha \bar{\gamma }\right)}^{2}}$, Lagrange multiplier is $\lambda^{N•}=\frac{\bar{\alpha }(\alpha -2\alpha \gamma -\left(2-\alpha \gamma \right)c_{n})+\left(2-\alpha \bar{\gamma }\right)s_{r}}{\left(1+\alpha \gamma )(2-\alpha \bar{\gamma }\right)}$.

### 3.2 P mode game model

In the P mode, after the government sets the recycling rate target, manufacturers do not make improvement measures and pay fines for the portion that falls below the target value.

The profit maximization function for the manufacturer is:

$$\begin{array}{c} \begin{array}{c} \text{max}\;\pi_{M}^{P}=(p_{n}-c_{n}-m\delta_{n})q_{n} \end{array} \end{array}$$ (8)

The profit maximization function for the remanufacturer is:

$$\begin{array}{c} \begin{array}{c} \text{max}\;\pi_{R}^{P}=(p_{r}-c_{n}+s_{r})q_{r}\\ s.t.q_{r}\leq \gamma q_{n} \end{array} \end{array}$$ (9)

Construct the Lagrange function:

$$\begin{array}{c} \begin{array}{c} L\left(p_{r},\lambda \right)=\pi_{R}^{P}+\lambda \left(\gamma q_{n}-q_{r}\right) \end{array} \end{array}$$ (10)

λ is the Lagrange multiplier, and λ ≥ 0.

The manufacturer decides the selling price of the new product *p*_*n*_, and the remanufacturer decides the selling price of the remanufactured product *p*_*r*_. Eq (8) is a concave function with respect to *p*_*n*_, while Eqs (9) and (10) are concave functions with respect to *p*_*r*_.

Manufacturer determines the sales price *p*_*n*_ for the new product, and the remanufacturer sets the sales price *p*_*r*_ for the remanufactured product. Eq (8) is a concave function with respect to *p*_*n*_, and Eqs (9) and (10) are concave functions with respect to *p*_*r*_. To achieve profit maximization, the following conditions must be satisfied: $\frac{\partial^{2}\pi_{M}^{P}}{\partial {p_{n}}^{2}}=-\frac{2}{\bar{\alpha }}<0$,$\frac{\partial^{2}\pi_{R}^{P}}{\partial {p_{r}}^{2}}=-\frac{2}{\alpha \bar{\alpha }}<0$,$\frac{\partial^{2}L\left(p_{r},\lambda \right)}{\partial {p_{r}}^{2}}=-\frac{2}{\alpha \bar{\alpha }}<0$,$\frac{\partial^{2}L\left(p_{r},\lambda \right)}{\partial {p_{r}}^{2}}=-\frac{2}{\alpha \bar{\alpha }}<0$.

Taking the first-order partial derivative of Eq (8) with respect to *p*_*n*_, we obtain:

$$\begin{array}{c} \begin{array}{c} \frac{\partial \pi_{M}^{P}}{\partial p_{n}}=\frac{\bar{\alpha }+c_{n}-2p_{n}+p_{r}+m\delta_{n}}{\bar{\alpha }} \end{array} \end{array}$$ (11)

1) When *γ* ≥ *γ*^P*^, λ = 0, indicating that remanufacturing is unrestricted.

Taking the first-order partial derivative of Eq (9) with respect to *p*_*r*_, we obtain:

$$\begin{array}{c} \begin{array}{c} \frac{\partial \pi_{R}^{P}}{\partial p_{r}}=\frac{c_{n}+\alpha p_{n}-2p_{r}-s_{r}}{\alpha \bar{\alpha }} \end{array} \end{array}$$ (12)

By setting Eqs (11) and (12) to zero and solving simultaneously, the optimal solution is obtained:

The selling price of new products is $p_{n}^{P*}=\frac{2\bar{\alpha }+3c_{n}-s_{r}+2m\delta_{n}}{3+\bar{\alpha }}$, the selling price of remanufactured products is $p_{r}^{P*}=\frac{\alpha \bar{\alpha }+(2+\alpha )c_{n}-2s_{r}+m\alpha \delta_{n}}{3+\bar{\alpha }}$, the sales volume of new products is $q_{n}^{P*}=\frac{2\bar{\alpha }-\bar{\alpha }c_{n}-s_{r}-m(1+\bar{\alpha })\delta_{n}}{(3+\bar{\alpha })\bar{\alpha }}$, the sales volume of remanufactured products is $q_{r}^{P*}=\frac{s_{r}+\bar{\alpha }(\alpha -2c_{n}+s_{r})+m\alpha \delta_{n}}{(3+\bar{\alpha })\alpha \bar{\alpha }}$, the profit of the manufacturer is $\pi_{M}^{P*}=\frac{{\left(\left(2-c_{n}\right)\bar{\alpha }-s_{r}-m(1+\bar{\alpha })\delta_{n}\right)}^{2}}{{\left(3+\bar{\alpha }\right)}^{2}\bar{\alpha }}$, the profit of the remanufacturer is $\pi_{R}^{P*}=\frac{{\left(s_{r}+\bar{\alpha }(\alpha -2c_{n}+s_{r})+m\alpha \delta_{n}\right)}^{2}}{{\left(3+\bar{\alpha }\right)}^{2}\alpha \bar{\alpha }}$, $\gamma^{P*}=\frac{q_{r}^{P*}}{q_{n}^{P*}}=\frac{s_{r}+\bar{\alpha }(\alpha -2c_{n}+s_{r})+m\alpha \delta_{n}}{\alpha (\left(2-c_{n}\right)\bar{\alpha }-s_{r}-m\delta_{n}\left(1+\bar{\alpha }\right))}$.

2) When *γ* < *γ*^P*^, λ ≠ 0, indicating that remanufacturing is restricted.

Taking the first-order partial derivatives of Eq (10) with respect to λ and *p*_*r*_, we obtain:

$$\begin{array}{c} \begin{array}{c} \frac{\partial L(p_{r},\lambda )}{\partial \lambda }=\frac{\alpha \gamma \bar{\alpha }-\alpha (1+\gamma )p_{n}+(1+\alpha \gamma )p_{r}}{\alpha \bar{\alpha }} \end{array} \end{array}$$ (13)

$$\begin{array}{c} \begin{array}{c} \frac{\partial L(p_{r},\lambda )}{\partial p_{r}}=\frac{\lambda +\alpha \gamma \lambda +c_{n}+\alpha p_{n}-2p_{r}-s_{r}}{\alpha \bar{\alpha }} \end{array} \end{array}$$ (14)

By setting Eqs (11), (13) and (14) to zero and solving simultaneously, the optimal solution is obtained:

The selling price of new products is $p_{n}^{P•}=\frac{\bar{\alpha }+(1+\alpha \gamma )(c_{n}+m\delta_{n})}{2-\alpha \bar{\gamma }}$, the selling price of remanufactured products is $p_{r}^{P•}=\frac{\alpha (\bar{\alpha }\bar{\gamma }+(1+\gamma )(c_{n}+m\delta_{n}))}{2-\alpha \bar{\gamma }}$, the sales volume of new products is $q_{n}^{P•}=\frac{{\bar{c}}_{n}-m\delta_{n}}{2-\alpha \bar{\gamma }}$, the sales volume of remanufactured products is $q_{r}^{P•}=\frac{\gamma ({\bar{c}}_{n}-m\delta_{n})}{2-\alpha \bar{\gamma }}$, the profit of the manufacturer is $\pi_{M}^{P•}=\frac{\bar{\alpha }{\left({\bar{c}}_{n}-m\delta_{n}\right)}^{2}}{{\left(2-\alpha \bar{\gamma }\right)}^{2}}$, the profit of the remanufacturer is $\pi_{R}^{P•}=\frac{\gamma ({\bar{c}}_{n}-m\delta_{n})(\bar{\alpha }(\alpha \bar{\gamma }-2c_{n})+(2-\alpha \bar{\gamma })s_{r}+m\alpha (1+\gamma )\delta_{n})}{{\left(2-\alpha \bar{\gamma }\right)}^{2}}$, Lagrange multiplier is $\lambda^{P•}=\frac{\bar{\alpha }(\alpha -2\alpha \gamma -(2-\alpha \gamma )c_{n})+(2-\alpha \bar{\gamma })s_{r}}{\left(1+\alpha \gamma )(2-\alpha \bar{\gamma }\right)}$.

### 3.3 E mode game model

In the E mode, after the government sets the recycling rate target, manufacturers actively take measures to improve the recycling rate of their products. After improvement, if the recycling rate exceeds the target value, the company receives a reward; if the recycling rate falls below the target value, the company pays a fine.

The profit maximization function for the manufacturer is:

$$\begin{array}{c} \begin{array}{c} \text{max}\;\pi_{M}^{E}=(p_{n}-c_{n}+\left(\tau -m\right)\delta_{n})q_{n}-k\tau^{2}/2 \end{array} \end{array}$$ (15)

The profit maximization function for the remanufacturer is:

$$\begin{array}{c} \begin{array}{c} \text{max}\;\pi_{R}^{E}=(p_{r}-c_{n}+s_{r}+\tau \delta_{r})q_{r}\\ s.t.q_{r}\leq \gamma q_{n} \end{array} \end{array}$$ (16)

Construct the Lagrange function:

$$\begin{array}{c} \begin{array}{c} L\left(p_{r},\lambda \right)=\pi_{R}^{E}+\lambda \left(\gamma q_{n}-q_{r}\right) \end{array} \end{array}$$ (17)

λ is the Lagrange multiplier, and λ ≥ 0.

First, the manufacturer decides the level of effort in eco-design, denoted by *τ*. Next, the manufacturer sets the selling price of the new product, *p*_*n*_, and the remanufacturer sets the selling price of the remanufactured product, *p*_*r*_. The solution is obtained using the backward induction method. Eq (15) is a concave function with respect to *p*_*n*_, while Eqs (16) and (17) are concave functions with respect to *p*_*r*_. To achieve profit maximization, the following conditions must be simultaneously satisfied: $\frac{\partial^{2}\pi_{M}^{E}}{\partial {p_{n}}^{2}}=-\frac{2}{\bar{\alpha }}<0$,$\frac{\partial^{2}\pi_{M}^{E}}{\partial \tau^{2}}=-k$,$\frac{\partial^{2}\pi_{M}^{E}}{\partial {p_{n}}^{2}}\frac{\partial^{2}\pi_{M}^{E}}{\partial \tau^{2}}-{\left(\frac{\partial^{2}\pi_{M}^{E}}{\partial p_{n}\partial \tau }\right)}^{2}=\frac{2k\bar{\alpha }-{\delta_{n}}^{2}}{{\bar{\alpha }}^{2}}>0$,$\frac{\partial^{2}\pi_{R}^{E}}{\partial {p_{r}}^{2}}=-\frac{2}{\alpha \bar{\alpha }}<0$,$\frac{\partial^{2}L\left(p_{r},\lambda \right)}{\partial {p_{r}}^{2}}=-\frac{2}{\alpha \bar{\alpha }}<0$,$\frac{\partial \pi_{M}^{E}}{\partial p_{n}}=0$,$\frac{\partial \pi_{R}^{E}}{\partial \tau }=0$,$\frac{\partial \pi_{R}^{E}}{\partial p_{r}}=0$.

Taking the first-order partial derivative of Eq (15) with respect to *p*_*n*_, we obtain:

$$\begin{array}{c} \begin{array}{c} \frac{\partial \pi_{M}^{E}}{\partial p_{n}}=\frac{\bar{\alpha }+c_{n}-2p_{n}+p_{r}+(m-\tau )\delta_{n}}{\bar{\alpha }} \end{array} \end{array}$$ (18)

1) When *γ* ≥ *γ*^E*^, λ = 0, remanufacturing is unrestricted.

Taking the first-order partial derivative of Eq (16) with respect to *p*_*r*_, we obtain:

$$\begin{array}{c} \begin{array}{c} \frac{\partial \pi_{R}^{E}}{\partial p_{r}}=\frac{c_{n}+\alpha p_{n}-2p_{r}-s_{r}-\tau \delta_{r}}{\alpha \bar{\alpha }} \end{array} \end{array}$$ (19)

By setting Eqs (18) and (19) to zero and solving simultaneously, the optimal solution is obtained: $p_{n}=\frac{2\bar{\alpha }+3c_{n}-s_{r}+2(m-\tau )\delta_{n}-\tau \delta_{r}}{3+\bar{\alpha }}$, $p_{r}=\frac{(2+\alpha )c_{n}-2s_{r}+\alpha (\bar{\alpha }+(m-\tau )\delta_{n})-2\tau \delta_{r}}{3+\bar{\alpha }}$.

Substituting the above optimal solutions into Eq (15), we obtain:

$$\begin{array}{c} \begin{array}{c} \pi_{M}^{E}=\frac{2{\left(\left(2-c_{n}\right)\bar{\alpha }-s_{r}+(\tau -m)(1+\bar{\alpha })\delta_{n}-\tau \delta_{r}\right)}^{2}-k{\left(3+\bar{\alpha }\right)}^{2}\tau^{2}\bar{\alpha }}{2{\left(3+\bar{\alpha }\right)}^{2}\bar{\alpha }} \end{array} \end{array}$$ (20)

In Eq (20), the coefficient of the quadratic term of *τ* is negative, indicating that $\pi_{M}^{E}$ is a concave function with respect to *τ*. Taking the first-order partial derivative of Eq (20) with respect to *τ* and setting it to zero, the optimal solution is obtained:

The level of eco-design is $\tau^{E*}=\frac{2(\left(2-c_{n}\right)\bar{\alpha }-s_{r}-m(1+\bar{\alpha })\delta_{n})((1+\bar{\alpha })\delta_{n}-\delta_{r})}{k{\left(3+\bar{\alpha }\right)}^{2}\bar{\alpha }-2{\left((1+\bar{\alpha })\delta_{n}-\delta_{r}\right)}^{2}}$, The selling price of new products is $p_{n}^{E*}=\frac{2\bar{\alpha }+3c_{n}-s_{r}+2(m-\tau^{E*})\delta_{n}-\delta_{r}\tau^{E*}}{3+\bar{\alpha }}$, the selling price of remanufactured products is $p_{r}^{E*}=\frac{(2+\alpha )c_{n}-2s_{r}+\alpha (\bar{\alpha }+(m-\tau^{E*})\delta_{n})-2\delta_{r}\tau^{E*}}{3+\bar{\alpha }}$, the sales volume of new products is $q_{n}^{E*}=\frac{\left(2-c_{n}\right)\bar{\alpha }-s_{r}-(m-\tau^{E*})(1+\bar{\alpha })\delta_{n}-\delta_{r}\tau^{E*}}{(3+\bar{\alpha })\bar{\alpha }}$, the sales volume of remanufactured products is $q_{r}^{E*}=\frac{s_{r}+\alpha (m-\tau^{E*})\delta_{n}+\delta_{r}\tau^{E*}+\bar{\alpha }(\alpha -2c_{n}+s_{r}+\delta_{r}\tau^{E*})}{(3+\bar{\alpha })\alpha \bar{\alpha }}$, the profit of the manufacturer is $\pi_{M}^{E*}=\frac{2{\left(\left(2-c_{n}\right)\bar{\alpha }-s_{r}-(m-\tau^{E*})(1+\bar{\alpha })\delta_{n}-\delta_{r}\tau^{E*}\right)}^{2}-k{\left(3+\bar{\alpha }\right)}^{2}{\left(\tau^{E*}\right)}^{2}\bar{\alpha }}{2{\left(3+\bar{\alpha }\right)}^{2}\bar{\alpha }}$, the profit of the remanufacturer is $\pi_{R}^{E*}=\frac{{\left(s_{r}+\alpha (m-\tau^{E*})\delta_{n}+\delta_{r}\tau^{E*}+\bar{\alpha }(\alpha -2c_{n}+s_{r}+\delta_{r}\tau^{E*})\right)}^{2}}{{\left(3+\bar{\alpha }\right)}^{2}\alpha \bar{\alpha }}$, $\gamma^{E*}=\frac{q_{r}^{E*}}{q_{n}^{E*}}=\frac{s_{r}+\alpha (m-\tau )\delta_{n}+\tau \delta_{r}+\bar{\alpha }(\alpha -2c_{n}+s_{r}+\tau \delta_{r})}{\alpha (\left(2-c_{n}\right)\bar{\alpha }-s_{r}-(m-\tau )(1+\bar{\alpha })\delta_{n}-\tau \delta_{r})}$.

2) When *γ* < *γ*^E*^, λ ≠ 0, remanufacturing is restricted.

Taking the first-order partial derivatives of Eq (17) with respect to λ and *p*_*r*_, we obtain:

$$\begin{array}{c} \begin{array}{c} \frac{\partial L(p_{r},\lambda )}{\partial \lambda }=\frac{\alpha \gamma \bar{\alpha }-\alpha (1+\gamma )p_{n}+(1+\alpha \gamma )p_{r}}{\alpha \bar{\alpha }} \end{array} \end{array}$$ (21)

$$\begin{array}{c} \begin{array}{c} \frac{\partial L(p_{r},\lambda )}{\partial p_{r}}=\frac{\lambda +\alpha \gamma \lambda +c_{n}+\alpha p_{n}-2p_{r}-s_{r}-\tau \delta_{r}}{\alpha \bar{\alpha }} \end{array} \end{array}$$ (22)

By setting Eqs (18), (21) and (22) to zero and solving them simultaneously, the optimal solution is obtained:

$p_{n}=\frac{\bar{\alpha }+(1+\alpha \gamma )(c_{n}+(m-\tau )\delta_{n})}{2-\alpha \bar{\gamma }}$ , $p_{r}=\frac{\alpha (\bar{\alpha }\bar{\gamma }+(1+\gamma )c_{n}+(1+\gamma )(m-\tau )\delta_{n})}{2-\alpha \bar{\gamma }}$.

Substituting the above solutions into Eq (15), taking the first-order partial derivative with respect to *τ*, and setting it to zero, the optimal solution is obtained:

The level of eco-design is $\tau^{E•}=\frac{2\bar{\alpha }\delta_{n}({\bar{c}}_{n}-m\delta_{n})}{k{\left(2-\alpha \bar{\gamma }\right)}^{2}-2\bar{\alpha }\delta_{n}^{2}}$, the selling price of new products is $p_{n}^{E•}=\frac{k(1+\alpha \gamma )(2-\alpha \bar{\gamma })(c_{n}+m\delta_{n})+\bar{\alpha }(2k-k\alpha \bar{\gamma }-2\delta_{n}^{2})}{k{\left(2-\alpha \bar{\gamma }\right)}^{2}-2\bar{\alpha }\delta_{n}^{2}}$, the selling price of remanufactured products is $p_{r}^{E•}=\frac{\alpha (k(1+\gamma )(2-\alpha \bar{\gamma })(c_{n}+m\delta_{n})+\bar{\alpha }(k\bar{\gamma }(2-\alpha \bar{\gamma })-2\delta_{n}^{2}))}{k{\left(2-\alpha \bar{\gamma }\right)}^{2}-2\bar{\alpha }\delta_{n}^{2}}$, the sales volume of new products is $q_{n}^{E•}=\frac{k(2-\alpha \bar{\gamma })({\bar{c}}_{n}-m\delta_{n})}{k{\left(2-\alpha \bar{\gamma }\right)}^{2}-2\bar{\alpha }\delta_{n}^{2}}$, the sales volume of remanufactured products is $q_{r}^{E•}=\frac{k\gamma (2-\alpha \bar{\gamma })({\bar{c}}_{n}-m\delta_{n})}{k{\left(2-\alpha \bar{\gamma }\right)}^{2}-2\bar{\alpha }\delta_{n}^{2}}$, the profit of the manufacturer is $\pi_{M}^{E•}=\frac{k\bar{\alpha }{\left({\bar{c}}_{n}-m\delta_{n}\right)}^{2}}{k{\left(2-\alpha \bar{\gamma }\right)}^{2}-2\bar{\alpha }\delta_{n}^{2}}$, the profit of the remanufacturer is $\pi_{R}^{E•}=\frac{\gamma ({\bar{c}}_{n}-\left(m-\tau^{G•}\right)\delta_{n})(\bar{\alpha }\left(\alpha \bar{\gamma }-2c_{n}\right)+\left(2-\alpha \bar{\gamma }\right)\left(s_{r}+\delta_{r}\tau^{G•}\right)+\alpha \left(1+\gamma \right)\left(m-\tau^{G•}\right)\delta_{n})}{{\left(2-\alpha \bar{\gamma }\right)}^{2}}$, the Lagrange multiplier is $\lambda^{E•}=\frac{\alpha \left(1-2\gamma \right)\bar{\alpha }-\left(2-\alpha \gamma \right)\bar{\alpha }c_{n}+\left(2-\alpha \bar{\gamma }\right)\left(s_{r}+\delta_{r}\tau^{G•}\right)+\alpha \left(m-\tau^{G•}\right)(1+\gamma (1+\bar{\alpha }))\delta_{n}}{\left(1+\alpha \gamma )(2-\alpha \bar{\gamma }\right)}$.

## 4 Analyze and compare equilibrium decisions

Analyze and compare the equilibrium decisions in three scenarios: before government regulation, after government regulation without improvement measures by the manufacturer, and after government regulation with the manufacturer adopting an eco-design strategy.

### 4.1 Trends of equilibrium decisions with changes in parameters

#### 4.1.1 The trend of equilibrium decision-making with changes in the recycling rate target

Proposition 1: In P-mode, the trend of equilibrium decision-making with changes in the recycling rate target is as follows:

(i) When remanufacturing is unrestricted (*γ* ≥ γ*), $\frac{\partial p_{n}^{P*}}{\partial m_{o}}>0$, $\frac{\partial p_{r}^{P*}}{\partial m_{o}}>0$, $\frac{\partial q_{n}^{P*}}{\partial m_{o}}<0$, $\frac{\partial q_{r}^{P*}}{\partial m_{o}}>0$,$\frac{\partial \pi_{M}^{P*}}{\partial m_{o}}<0$, $\frac{\partial \pi_{R}^{P*}}{\partial m_{o}}>0$.
(ii) When remanufacturing is restricted(*γ* < γ*), $\frac{\partial p_{n}^{P•}}{\partial m_{o}}>0$, $\frac{\partial p_{r}^{P•}}{\partial m_{o}}>0$, $\frac{\partial q_{n}^{P•}}{\partial m_{o}}<0$, $\frac{\partial q_{r}^{P•}}{\partial m_{o}}<0$, $\frac{\partial \pi_{M}^{P•}}{\partial m_{o}}<0$, $\frac{\partial^{2}\pi_{R}^{P•}}{\partial {m_{o}}^{2}}<0$.

Further mathematical derivations are provided in S1 File.

Proposition 2: In E-mode, the trend of equilibrium decision-making with changes in the recycling rate target is as follows:

(i) When remanufacturing is unrestricted (*γ* ≥ γ*), $\frac{\partial \tau^{E*}}{\partial m_{o}}<0$, $\frac{\partial p_{n}^{E*}}{\partial m_{o}}>0$, $\frac{\partial p_{r}^{E*}}{\partial m_{o}}>0$, $\frac{\partial q_{n}^{E*}}{\partial m_{o}}<0$, $\frac{\partial q_{r}^{E*}}{\partial m_{o}}>0$, $\frac{\partial \pi_{M}^{E*}}{\partial m_{o}}<0$, $\frac{\partial \pi_{R}^{E*}}{\partial m_{o}}>0$.
(ii) When remanufacturing is restricted(*γ* < γ*), $\frac{\partial \tau^{E•}}{\partial m_{o}}<0$, $\frac{\partial p_{n}^{E•}}{\partial m_{o}}>0$, $\frac{\partial p_{r}^{E•}}{\partial m_{o}}>0$, $\frac{\partial q_{n}^{E•}}{\partial m_{o}}<0$, $\frac{\partial q_{r}^{E•}}{\partial m_{o}}<0$, $\frac{\partial \pi_{M}^{E•}}{\partial m_{o}}<0$, $\frac{\partial^{2}\pi_{R}^{E•}}{\partial {m_{o}}^{2}}<0$.

Further mathematical derivations are provided in S1 File.

Based on Proposition 1 (i) and Proposition 2 (i), we conclude that that when remanufacturing is unrestricted, the trends in equilibrium decision changes with respect to recycling rate target are the same in both the E-mode and P-mode. As the target for recycling rates increases, the price of new products rises, and manufacturers transfer the penalty costs to consumers. The increase in the price of new products leads to a decline in their sales, resulting in reduced profits for manufacturers. The increase in new product prices drives up the prices of remanufactured products, with the rise in remanufactured product prices being lower than that of new products (as demonstrated in the S1 File). As a result, the sales volume of remanufactured products increases, and the gains from this higher sales volume outweigh the losses caused by the price increase, leading to higher profits for remanufacturers. The level of eco-design decreases as the recycling rate target increases. Raising the recycling rate target is detrimental to consumers, unfavorable for manufacturers, and does not contribute to enhancing eco-design levels; however, it is beneficial for remanufacturers.

Based on Proposition 1 (ii) and Proposition 2 (ii), we conclude that that when remanufacturing is restricted, the trends in equilibrium decision changes with respect to recycling rate targets are the same in both the E-mode and P-mode. As the recycling rate target increases, the price of new products rises. Manufacturers pass the penalty costs onto consumers. The increase in new product prices leads to a decrease in sales volume. Consequently, manufacturer profits decline. The increase in new product prices drives up the prices of remanufactured products. However, due to constraints on remanufacturing, the sales volume of remanufactured products decreases. Initially, the price increase boosts remanufacturer profits, but as the sales volume of remanufactured products decreases, the negative impact of declining sales volume on profits outweighs the positive impact of the price increase, resulting in remanufacturer profits initially rising and then falling.

Based on Propositions 1 and 2, in both P-mode and E-mode, regardless of whether remanufacturing is restricted, as the recycling rate target increases, the prices of new and remanufactured products rise. Manufacturers pass on the penalty costs to consumers, which is disadvantageous for consumers. The sales volume of new products decreases, and the manufacturer’s profit declines, which is unfavorable for manufacturers. When remanufacturing is unrestricted, as the recycling rate target increases, the sales volume of remanufactured products rises, leading to increased profits for remanufacturers, which is beneficial for them. However, when remanufacturing is restricted, the sales volume of remanufactured products decreases, and remanufacturers’ profits initially rise but then decline, meaning a higher recycling rate target does not necessarily benefit remanufacturers. Regardless of whether remanufacturing is restricted, the level of eco-design decreases as the recycling rate target increases; a lower recycling rate target is more conducive to enhancing eco-design.

#### 4.1.2 The trend of equilibrium decision-making with changes in the reward-penalty coefficient

Proposition 3: In P-mode, the trend of equilibrium decision-making with changes in the reward-penalty coefficient is as follows:

(i) When the remanufacturing is unrestricted (*γ* ≥ γ*), $\frac{\partial p_{n}^{P*}}{\partial \delta_{n}}>0$, $\frac{\partial p_{r}^{P*}}{\partial \delta_{n}}>0$, $\frac{\partial q_{n}^{P*}}{\partial \delta_{n}}<0$, $\frac{\partial q_{r}^{P*}}{\partial \delta_{n}}>0$, $\frac{\partial \pi_{M}^{P*}}{\partial \delta_{n}}<0$, $\frac{\partial \pi_{R}^{P*}}{\partial \delta_{n}}>0$.
(ii) When the remanufacturing is restricted (*γ* < γ*), $\frac{\partial p_{n}^{P•}}{\partial \delta_{n}}>0$, $\frac{\partial p_{r}^{P•}}{\partial \delta_{n}}>0$, $\frac{\partial q_{n}^{P•}}{\partial \delta_{n}}<0$, $\frac{\partial q_{r}^{P•}}{\partial \delta_{n}}<0$, $\frac{\partial \pi_{M}^{P•}}{\partial \delta_{n}}<0$, $\frac{\partial^{2}\pi_{R}^{P•}}{\partial {\delta_{n}}^{2}}<0$.

Further mathematical derivations are provided in S1 File.

Based on Propositions 1 and 3, in P-mode, the trend of equilibrium decision-making with changes in the reward-penalty coefficient is the same as that with changes in the recycling rate target.

Based on Proposition 3 (i), when remanufacturing is unrestricted, as the reward-penalty coefficient increases, the price of new products rises. Manufacturers pass the penalty costs onto consumers. The increase in new product prices leads to a decrease in sales volume. Consequently, manufacturer profits decline. The rise in new product prices drives up the prices of remanufactured products, but the increase in remanufactured product prices is less than that of new products (as proven in the S1 File), resulting in an increase in sales volume of remanufactured products. The benefits gained from the increased sales volume of remanufactured products outweigh the losses from the price increase, leading to higher profits for remanufacturers. Increasing the reward-penalty coefficient is unfavorable for consumers and manufacturers but beneficial for remanufacturers.

Based on Proposition 3 (ii), when remanufacturing is restricted, as the reward-penalty coefficient increases, the prices of new products rise, sales volume decreases, and manufacturer profits decline.The increase in new product prices drives up the prices of remanufactured products. However, due to constraints on remanufacturing, the sales volume of remanufactured products decreases. Initially, the price increase boosts remanufacturer profits, but as the sales volume of remanufactured products decreases, the negative impact of declining sales volume on profits outweighs the positive impact of the price increase, resulting in remanufacturer profits initially rising and then falling.

In E-mode, the trend of equilibrium decision-making with changes in the reward-penalty coefficient is related to the value range of *δ*_*n*_. This paper determines the value range of *δ*_*n*_ based on the constraints and uses simulation to investigate the trend of equilibrium decision-making in relation to changes in the reward-penalty coefficient.

### 4.2 Comparison of equilibrium decisions

#### 4.2.1 Comparison of equilibrium decisions between N-mode and P-mode

Proposition 4 (i): When remanufacturing is unrestricted, the comparison of equilibrium decisions between P-mode and N-mode is as follows:

$p_{n}^{P*}-p_{n}^{N*}>0$ ,$p_{r}^{P*}-p_{r}^{N*}>0$, $q_{n}^{P*}-q_{n}^{N*}<0$,$q_{r}^{P*}-q_{r}^{N*}>0$, $\pi_{M}^{P*}-\pi_{M}^{N*}<0$, $\pi_{R}^{P*}-\pi_{R}^{N*}>0$.

Proposition 4 (ii): When remanufacturing is restricted, the comparison of equilibrium decisions between P-mode and N-mode is as follows:

$p_{n}^{P•}-p_{n}^{N•}>0$ , $p_{r}^{P•}-p_{r}^{N•}>0$, $q_{n}^{P•}-q_{n}^{N•}<0$, $q_{r}^{P•}-q_{r}^{N•}<0$, $\pi_{M}^{P•}-\pi_{M}^{N•}<0$, $\pi_{R}^{P•}-\pi_{R}^{N•}>0$.

Further mathematical derivations are provided in S1 File.

Based on Proposition 4, after government sets the recycling rate target, regardless of whether remanufacturing is restricted, if manufacturers do not adopt improvement measures, the prices of new products and remanufactured products will rise, and manufacturers will pass the penalty costs onto consumers, which is unfavorable for consumers. The increase in new product prices leads to a decrease in sales volume and a decline in manufacturer profits. When remanufacturing is unrestricted, the sales volume of remanufactured products increases, resulting in higher profits for remanufacturers. Conversely, when remanufacturing is restricted, the sales volume of remanufactured products decreases, but the losses incurred from the decline in sales are lower than the gains from the price increase, so remanufacturer profits still rise.

#### 4.2.2 Comparison of equilibrium decisions between P-mode and E-mode

Proposition 5 (i): When remanufacturing is unrestricted, the comparison of equilibrium decisions between P-mode and N-mode is as follows:

$p_{n}^{E*}-p_{n}^{P*}<0$ , $p_{r}^{E*}-p_{r}^{P*}<0$,$q_{n}^{E*}-q_{n}^{P*}>0$, $q_{r}^{E*}-q_{r}^{P*}>0$, $\pi_{M}^{E*}-\pi_{M}^{P*}>0$, $\pi_{R}^{E*}-\pi_{R}^{P*}>0$.

Proposition 5 (ii): When remanufacturing is restricted, the comparison of equilibrium decisions between P-mode and N-mode is as follows:

$p_{n}^{E•}-p_{n}^{P•}<0$ , $p_{r}^{E•}-p_{r}^{P•}<0$, $q_{n}^{E•}-q_{n}^{P•}>0$, $q_{r}^{E•}-q_{r}^{P•}>0$, $\pi_{M}^{E•}-\pi_{M}^{P•}>0$, $\pi_{R}^{E•}-\pi_{R}^{P•}>0$.

Further mathematical derivations are provided in S1 File.

Based on Proposition 5, after government sets the recycling rate target, if manufacturers adopt eco-design, the prices of new products and remanufactured products will decrease. Eco-design is beneficial for consumers. With the reduction in prices for both new and remanufactured products, sales volumes for both will increase. Eco-design helps expand the market share of both types of products. Profits for both manufacturers and remanufacturers rise, making eco-design advantageous for both. Therefore, when government sets the recycling rate target, manufacturers would be well-advised to adopt eco-design to enhance the level of product recycling.

#### 4.2.3 Comparison of equilibrium decisions between N-mode and E-mode

Proposition 6 (i): When remanufacturing is unrestricted, the comparison of equilibrium decisions among N-mode, E-mode, and P-mode is as follows:

$q_{r}^{E*}>q_{r}^{P*}>q_{r}^{N*}$ , $\pi_{R}^{E*}>\pi_{R}^{P*}>\pi_{R}^{N*}$.

The comparison of the remaining equilibrium decisions in N-mode and E-mode is related to the values of *m*_*o*_ and *δ*_*n*_. Simulation is used to explore the comparison of these remaining equilibrium decisions.

Proposition 6 (ii): When remanufacturing is restricted, the comparison of equilibrium decisions among N-mode, E-mode, and P-mode is as follows:

$\pi_{R}^{E•}>\pi_{R}^{P•}>\pi_{R}^{N•}$ .

The comparison of the remaining equilibrium decisions in N-mode and E-mode is related to the values of *m*_*o*_ and *δ*_*n*_. Simulation is used to explore the comparison of these remaining equilibrium decisions.

Based on Proposition 4 (i) and Proposition 5 (i), we conclude that that when remanufacturing is unrestricted, $q_{r}^{E*}>q_{r}^{P*}>q_{r}^{N*}$, $\pi_{R}^{E*}>\pi_{R}^{P*}>\pi_{R}^{N*}$. Based on Proposition 4 (ii) and Proposition 5 (ii), we conclude that that when remanufacturing is restricted, $\pi_{R}^{E•}>\pi_{R}^{P•}>\pi_{R}^{N•}$.

## 5 Simulation

Using simulation to analyze the impact of the recycling rate target and reward-penalty coefficient on equilibrium decision-making. The equilibrium decision-making in N-mode is not affected by the recycling rate target and reward-penalty coefficient. This paper only discusses the trends in equilibrium decision-making in P-mode and E-mode with changes in the recycling rate target and reward-penalty coefficient.

### 5.1 Impact of recycling rate target on equilibrium decisions

**Fig 4:**
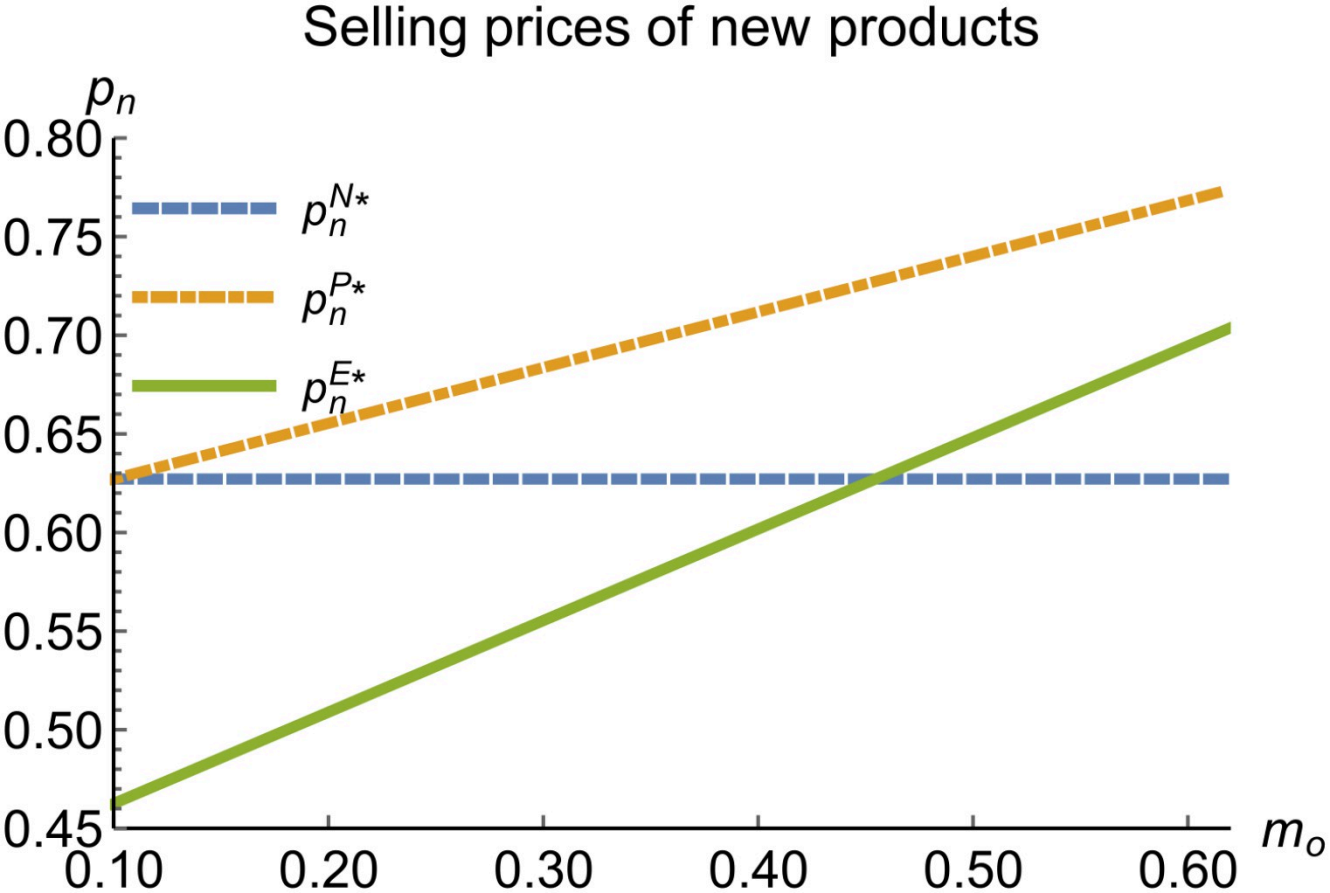
Selling prices of new products vary with *m*_*o*_.

**Fig 5:**
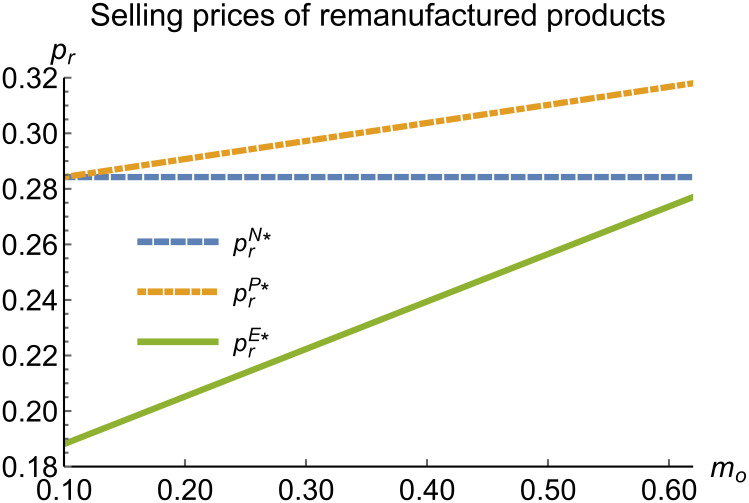
Selling prices of remanufactured products vary with *m*_*o*_.

**Fig 6:**
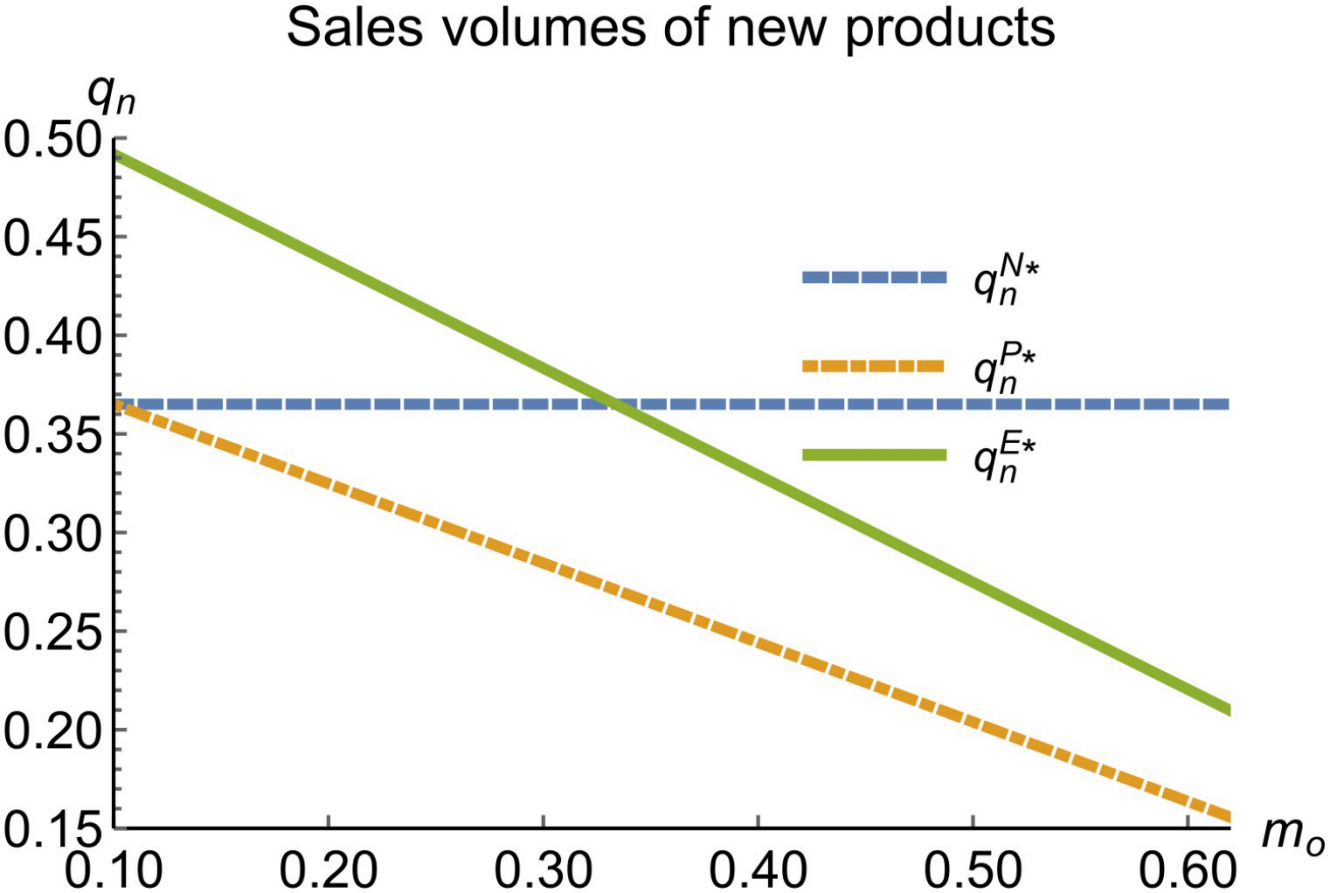
Sales volumes of new products vary with *m*_*o*_.

**Fig 7:**
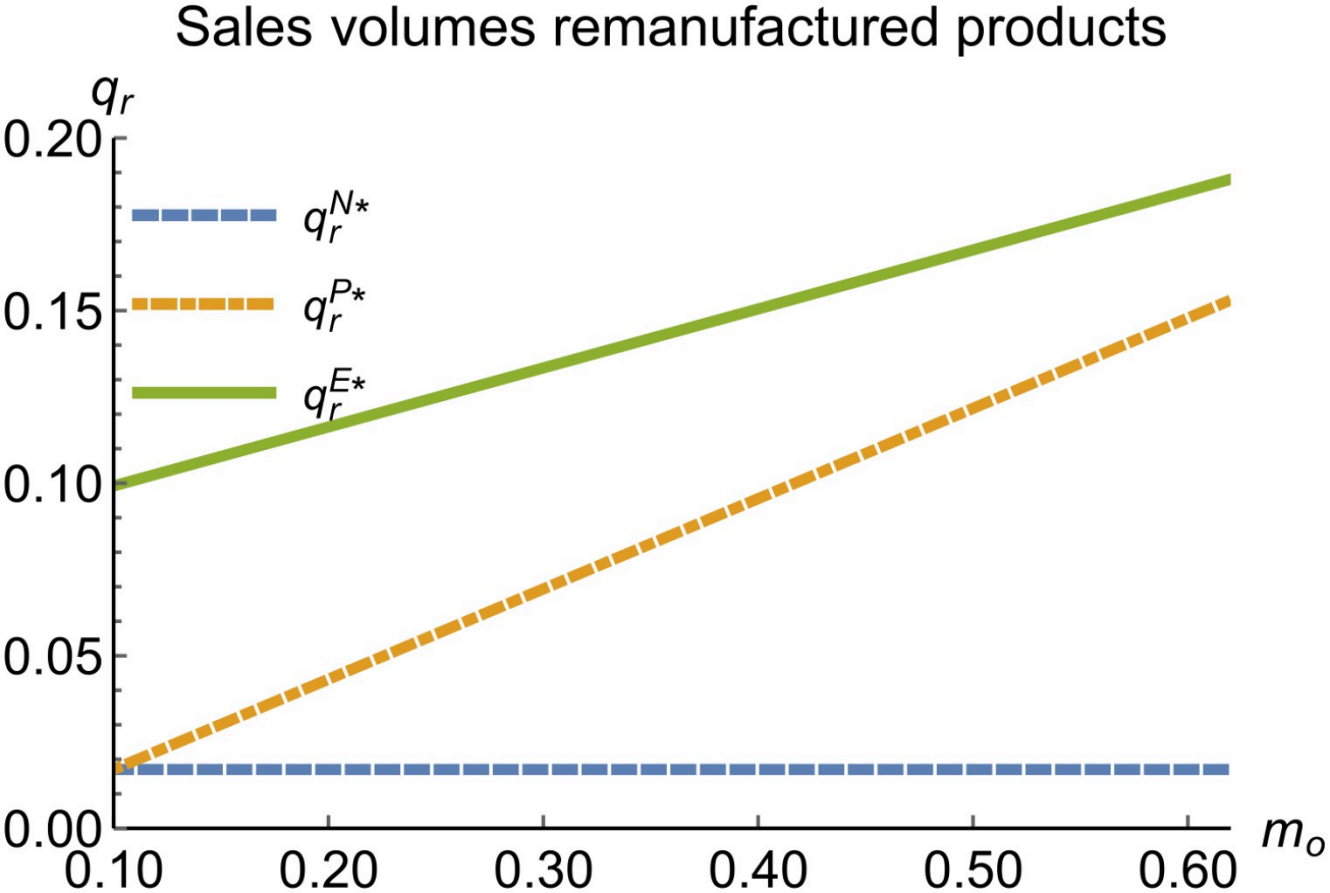
Sales volumes of remanufactured products vary with *m*_*o*_.

**Fig 8:**
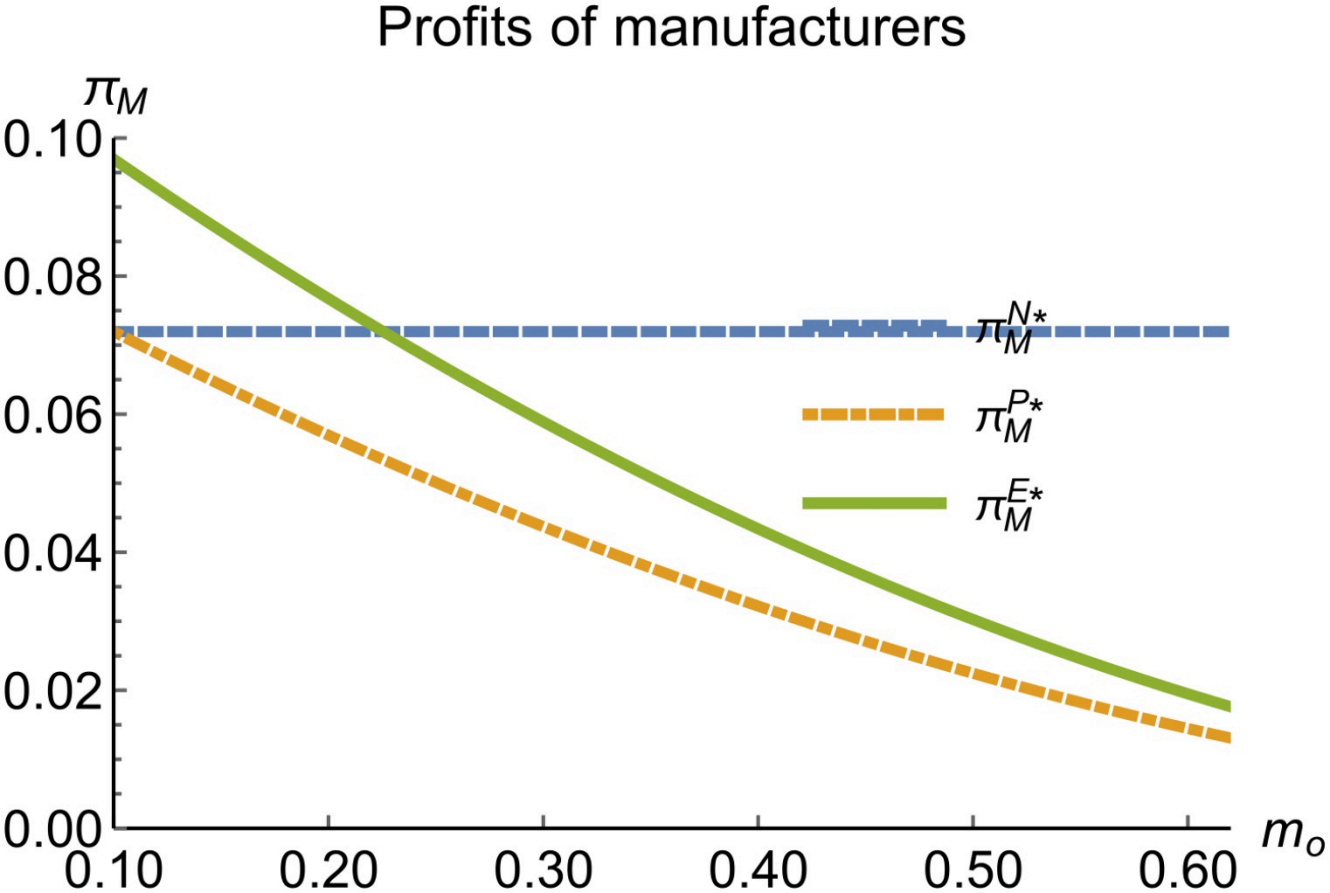
Profits of manufacturers vary with *m*_*o*_.

**Fig 9:**
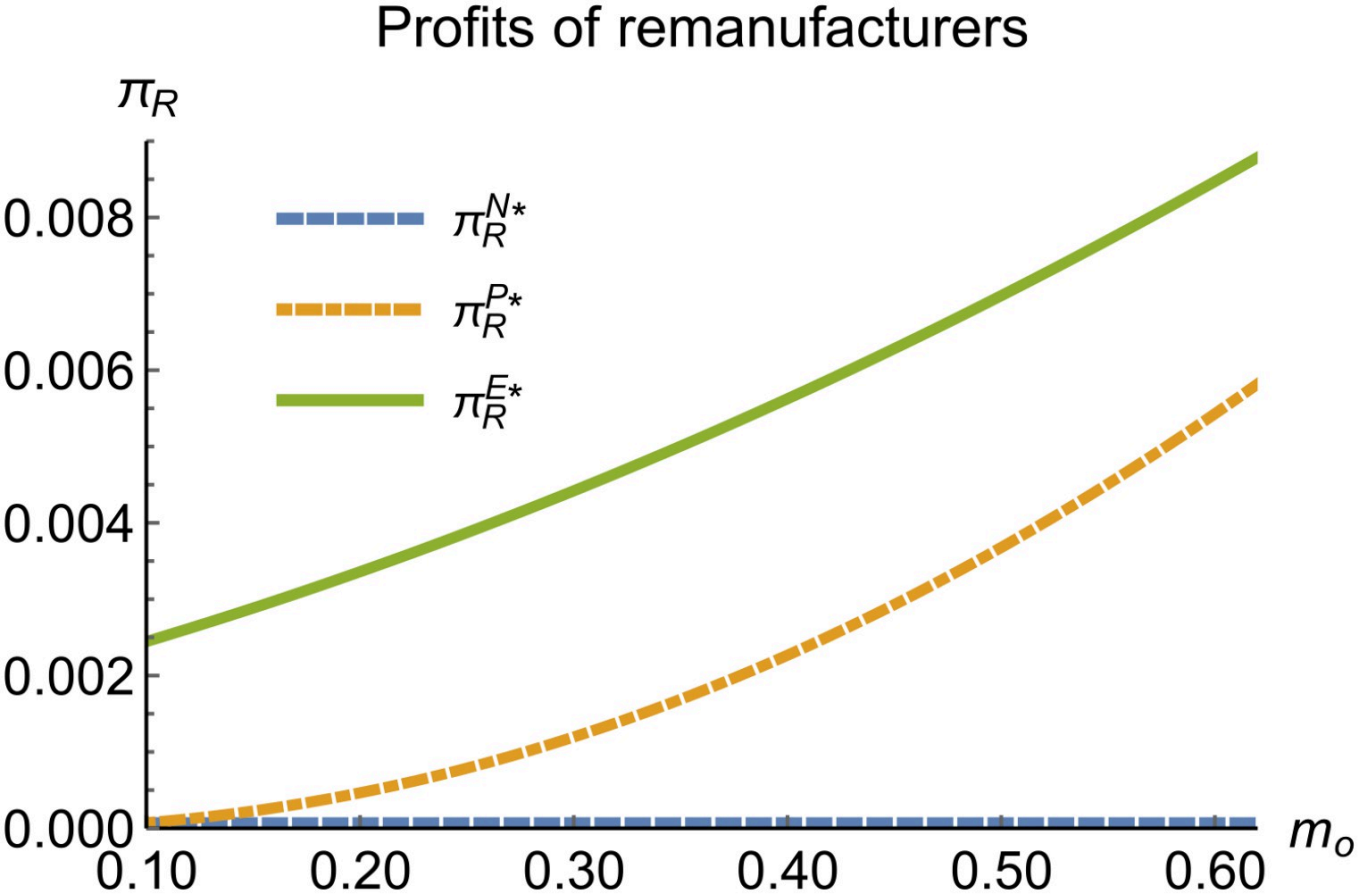
Profits of remanufacturers vary with *m*_*o*_.

**Fig 10:**
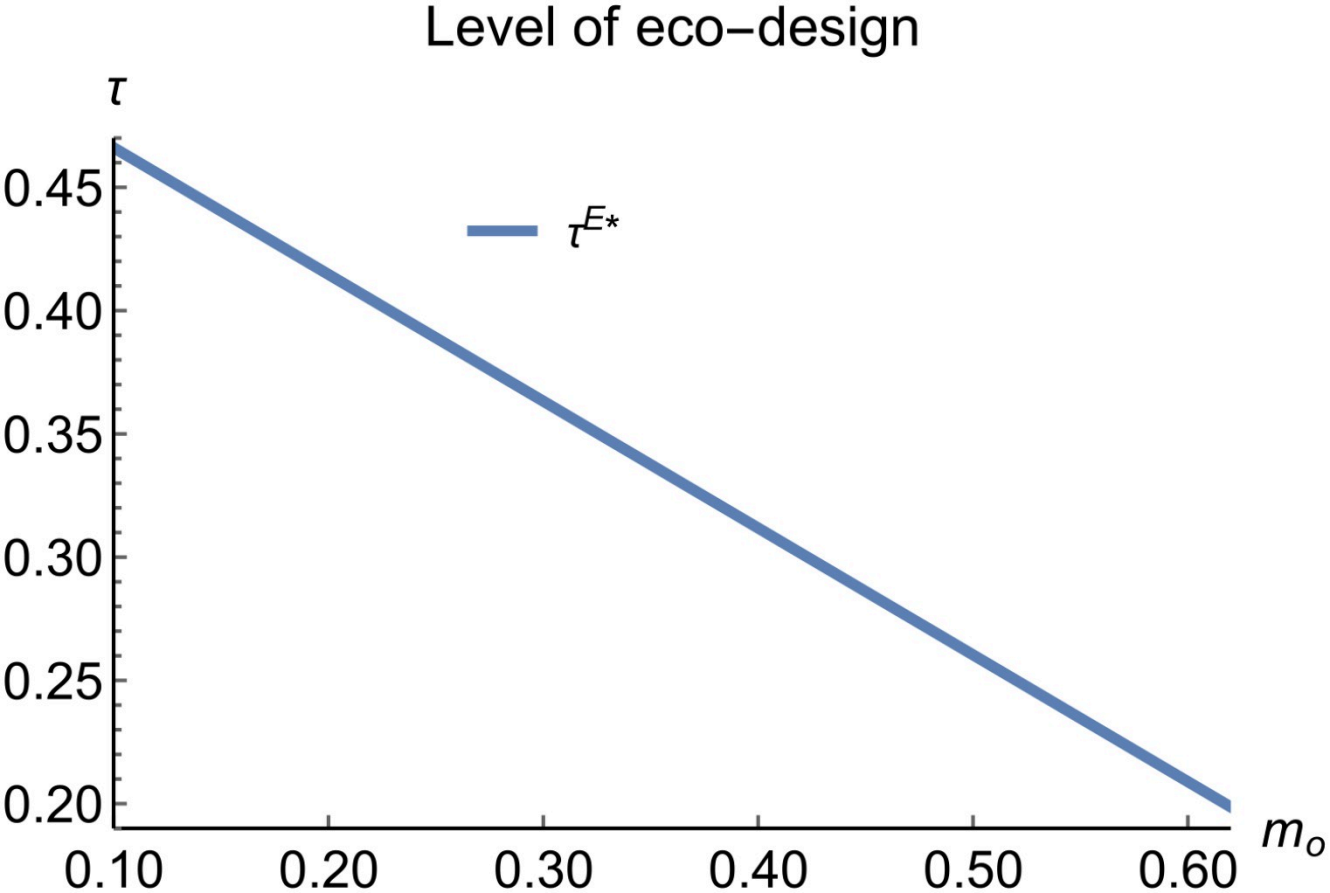
Level of eco-design vary with *m*_*o*_.

When remanufacturing is unrestricted, we discuss the trend of equilibrium decisions as the recycling rate target varies. The parameter assignments are shown in Table 2. To ensure that 0 < *p*_*n*_ < 1,0 < *p*_*r*_ < 1, 0 < *q*_*n*_,0 < *q*_*r*_, 0 < *π*_*M*_, 0 < *π*_*R*_, 0 < γ* < 1, 0 < *τ* < 1, *m*_0_ − *m*_*p*_ > 0 in three modes, *m*_0_ is set to (0.10, 0.62). The simulation results are illustrated in Figs 4–10. The trend of equilibrium decisions in Mode P is consistent with Proposition 1 (i), while the trend in Mode E aligns with Proposition 2 (i).

**Table 2:** Parameter assignment.

parameter	*m*_*p*_	*α*	*c*_*n*_	*s*_*r*_	*k*	*δ*_*n*_	*δ*_*r*_
assignment	0.1	0.46	0.43	0.15	0.31	0.5	0.25

**Fig 11:**
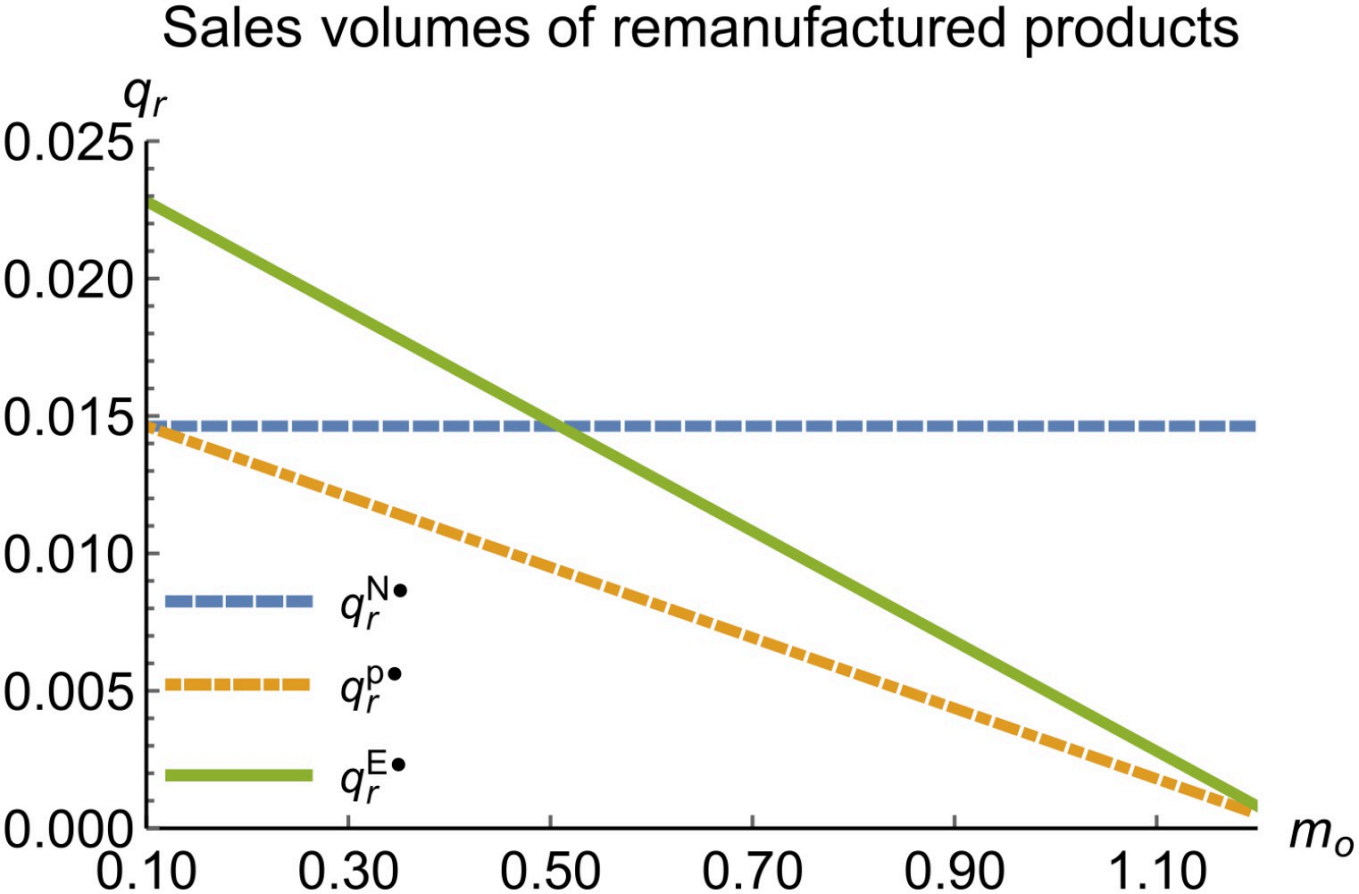
Sales volumes of remanufactured products vary with *m*_*o*_.

**Fig 12:**
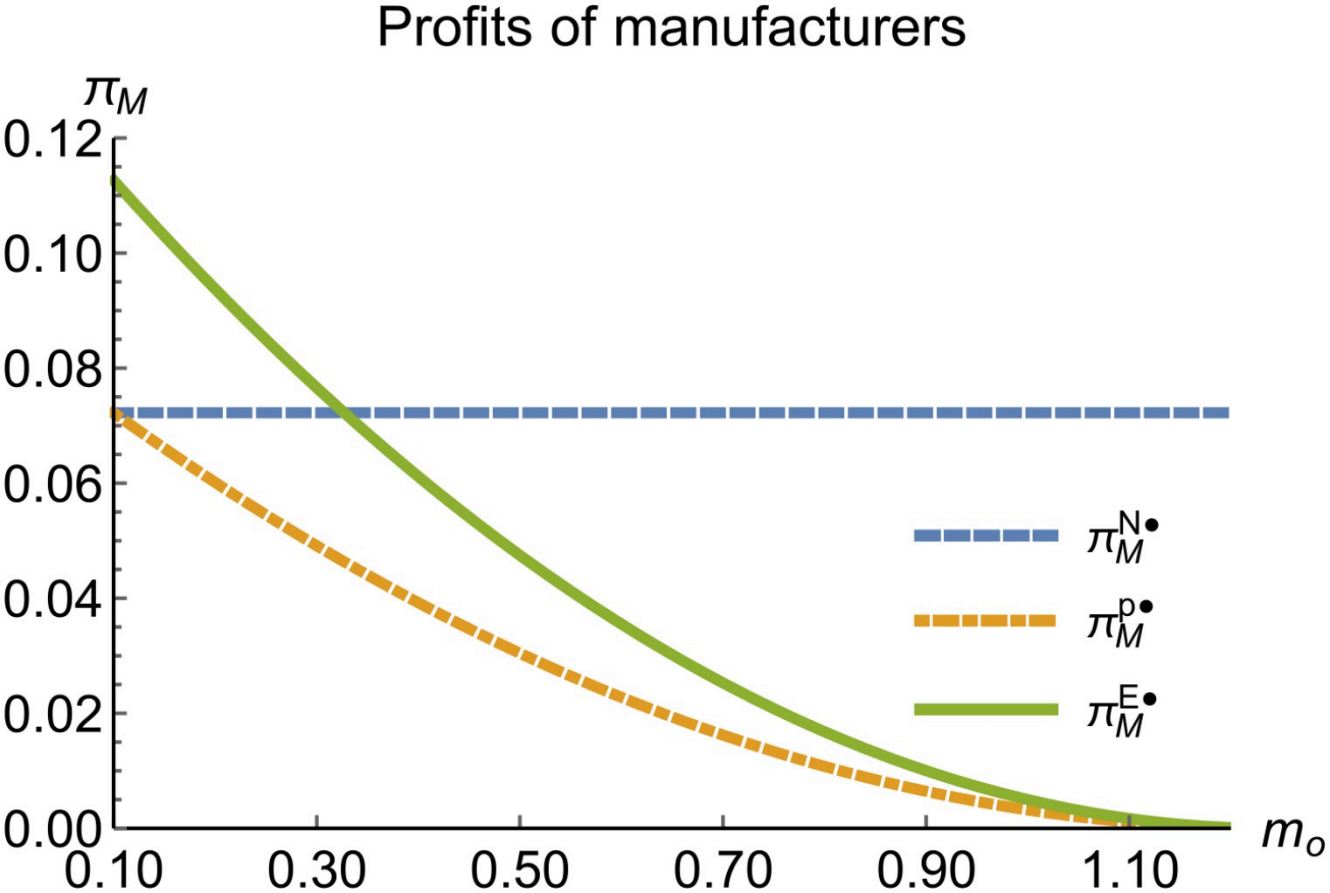
Profits of manufacturers vary with *m*_*o*_.

**Fig 13:**
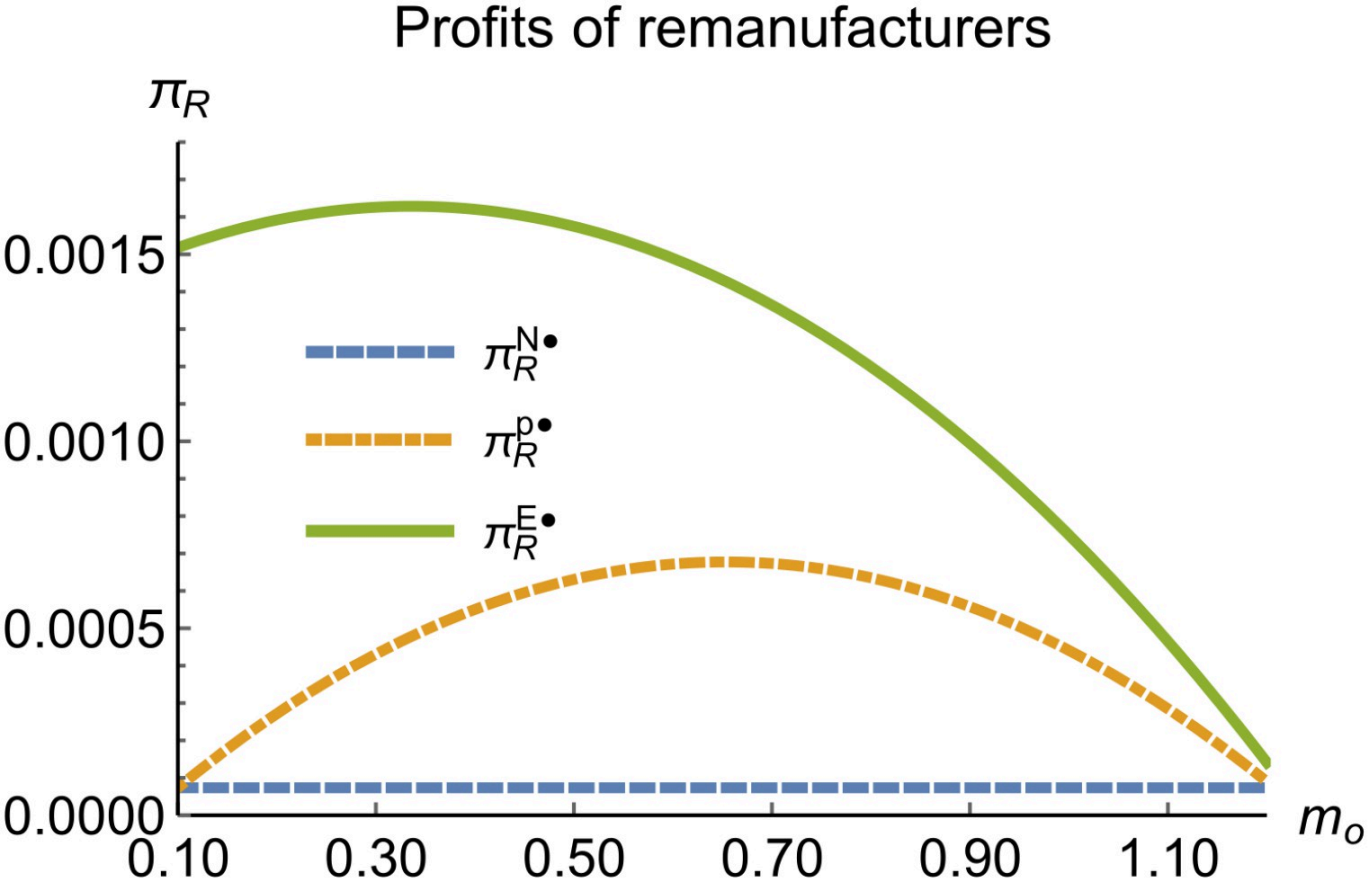
Profits of remanufacturers vary with *m*_*o*_.

When remanufacturing is constrained, we discuss the trends in equilibrium decisions as they change with the recycling rate target. Let *γ* = 0.04, with the remaining parameters assigned as shown in Table 2. To ensure that 0 < *p*_*n*_ < 1,0 < *p*_*r*_ < 1, 0 < *q*_*n*_, 0 < *q*_*r*_, 0 < *π*_*M*_, 0 < *π*_*R*_, *γ* < γ*, 0 < *τ* < 1,*m*_0_ − *m*_*p*_ > 0 in three modes, we set *m*_0_ to (0.10, 1.24). The simulation results indicate that the trend of equilibrium decision changes in the P mode is consistent with Proposition 1 (ii), while the trend in the E mode aligns with Proposition 2 (ii). This paper only presents the trend graphs of remanufactured product sales, manufacturer profits, and remanufacturer profits as they vary with recycling rate target, as shown in Figs 11–13; other figures are omitted.

### 5.2 Impact of reward-penalty coefficient on equilibrium decisions

**Fig 14:**
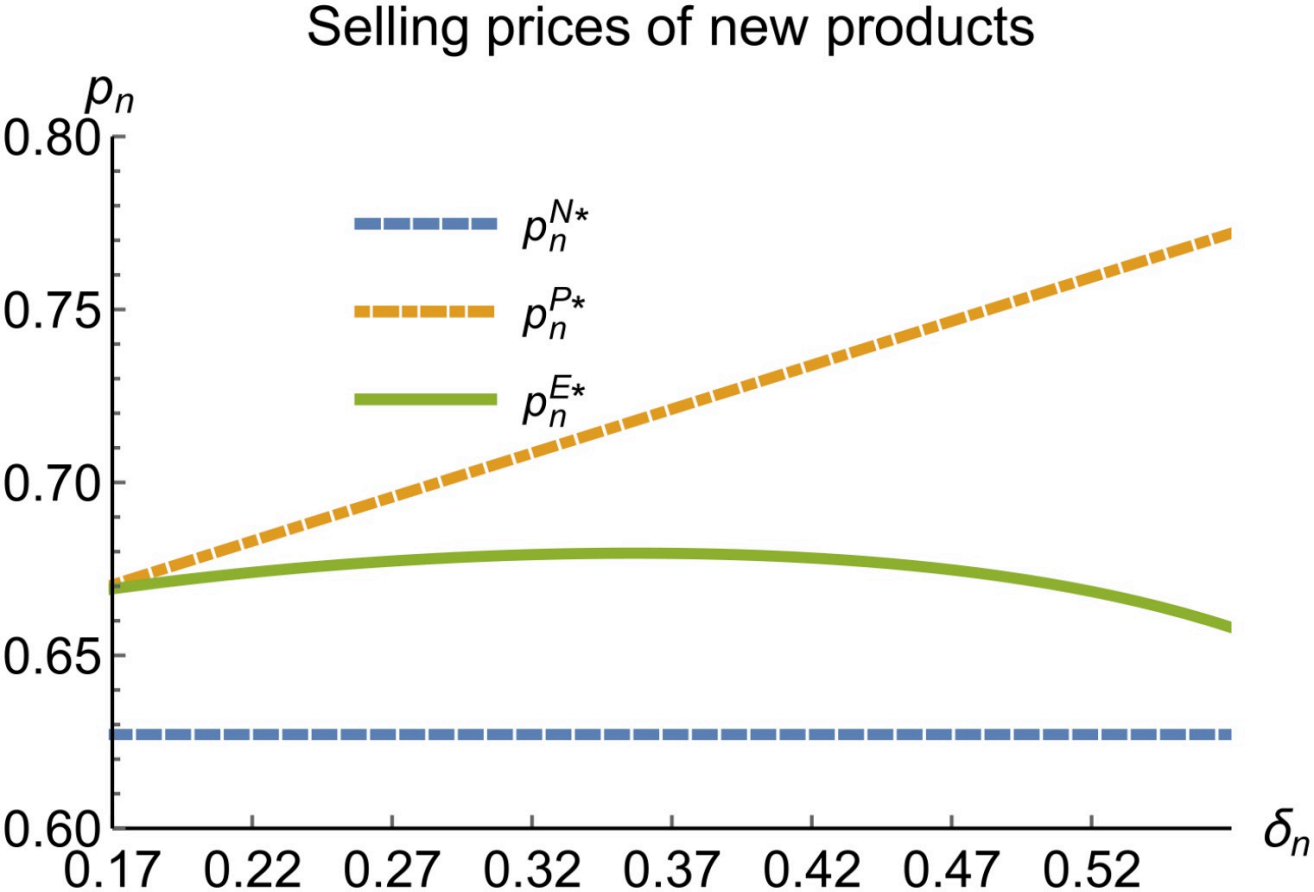
Selling prices of new products vary with *δ*_*n*_.

**Fig 15:**
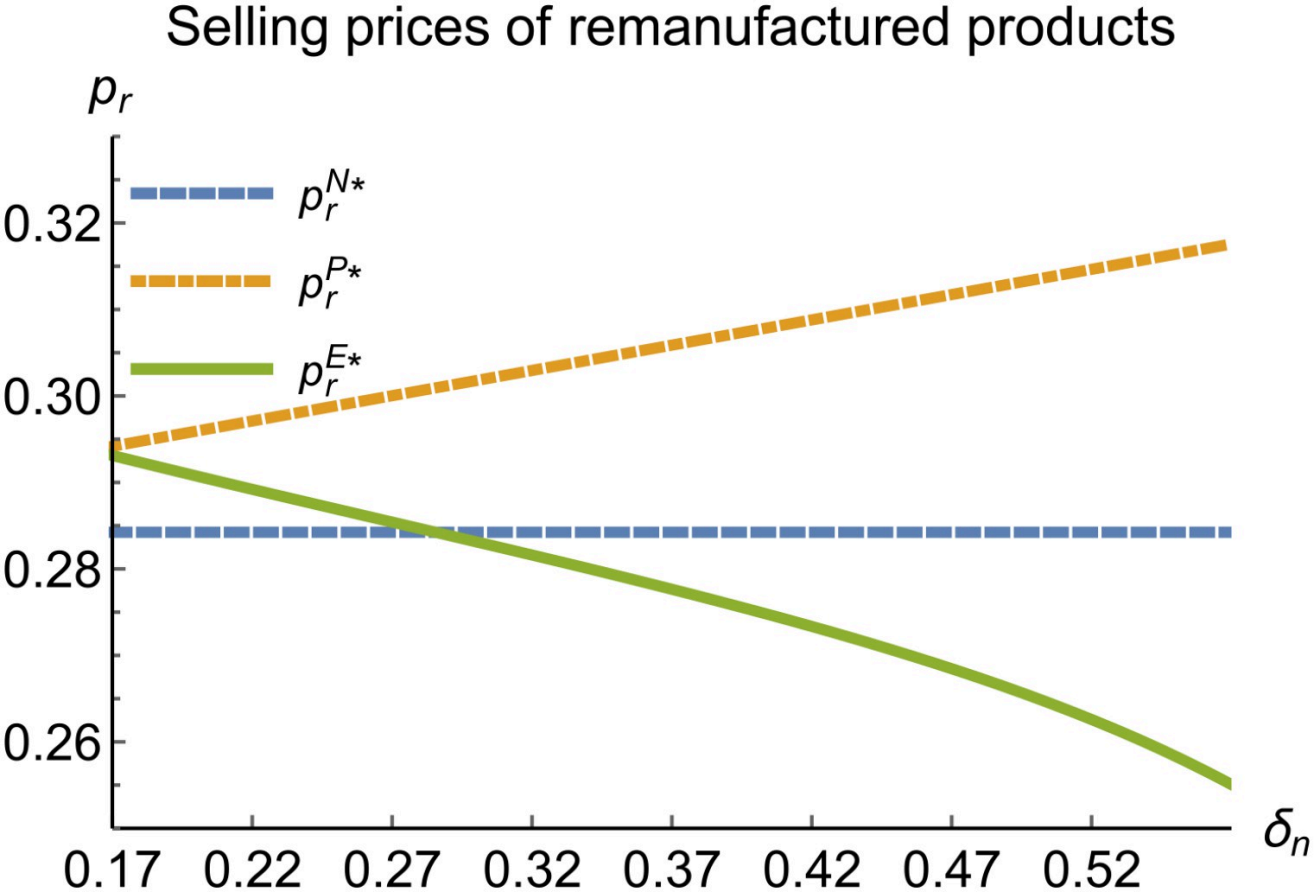
Selling prices of remanufactured products vary with *δ*_*n*_.

**Fig 16:**
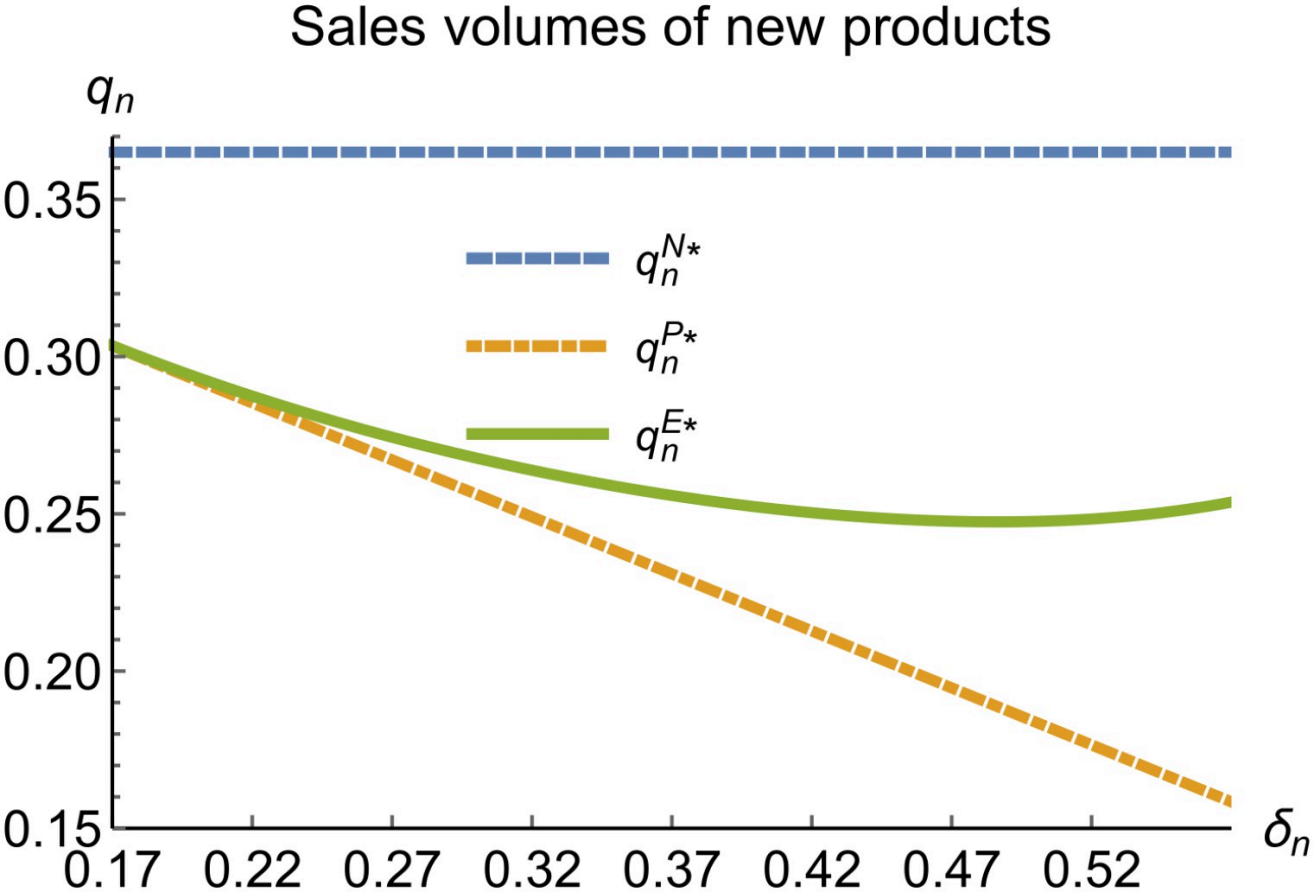
Sales volumes of new products vary with *δ*_*n*_.

**Fig 17:**
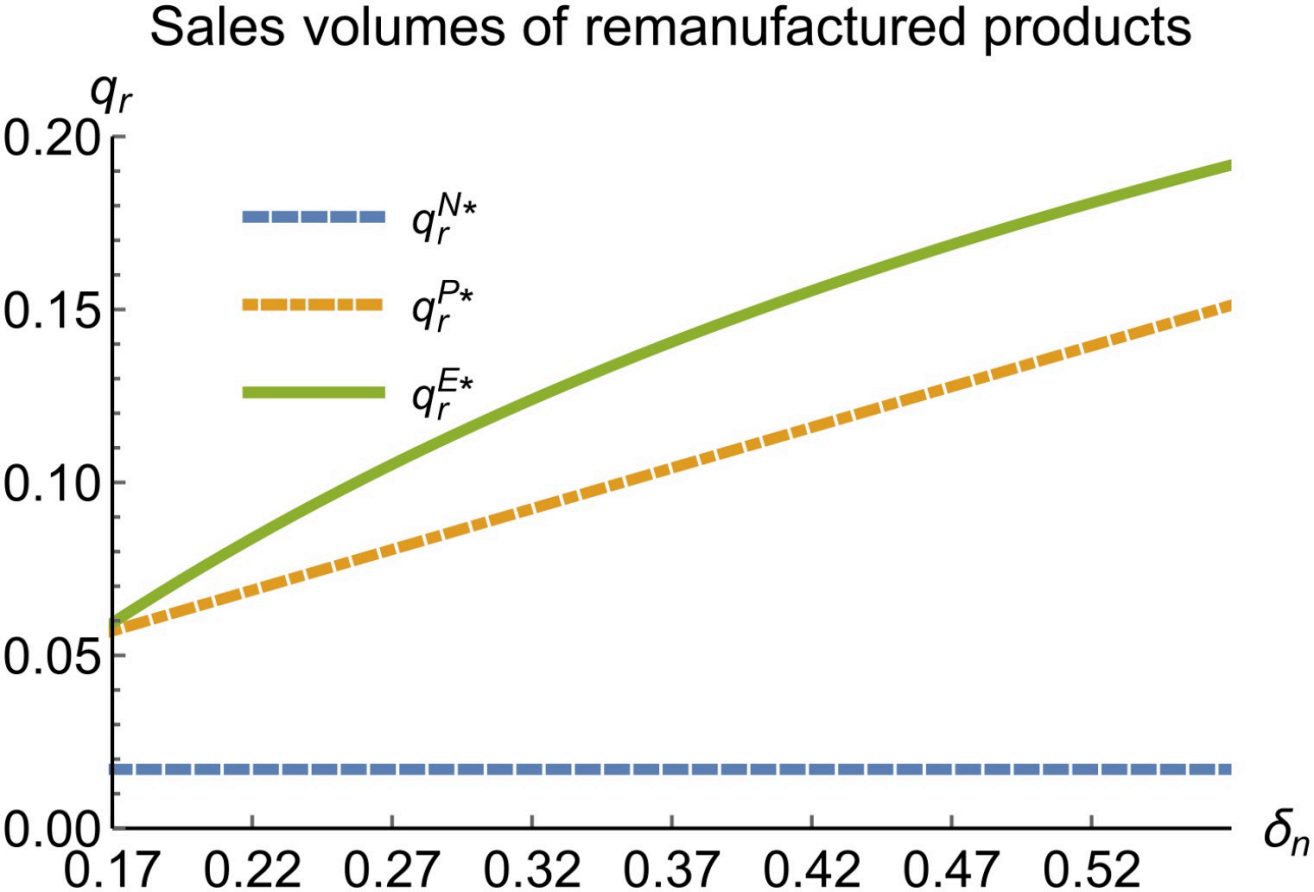
Sales volumes of remanufactured products vary with *δ*_*n*_.

**Fig 18:**
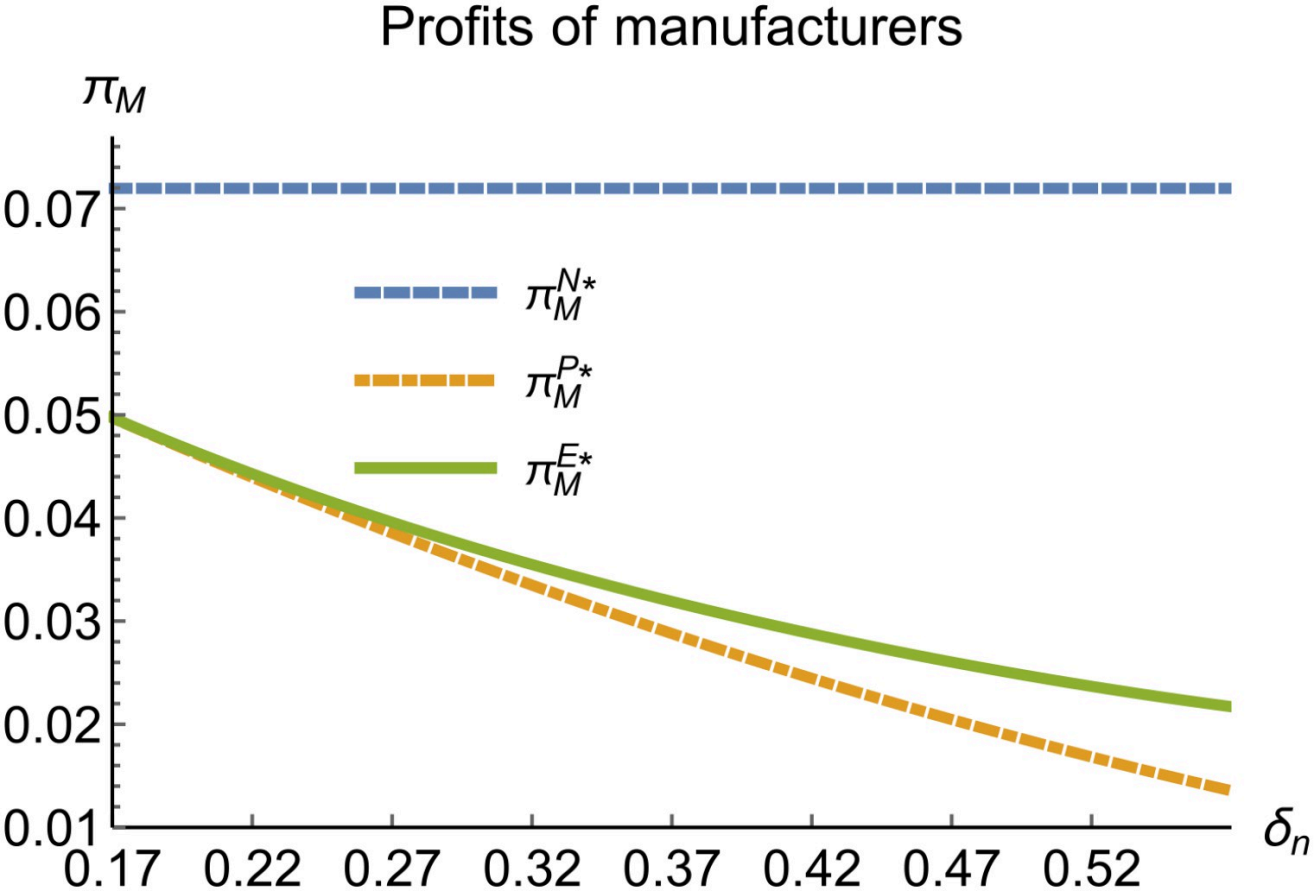
Profits of manufacturers vary with *δ*_*n*_.

**Fig 19:**
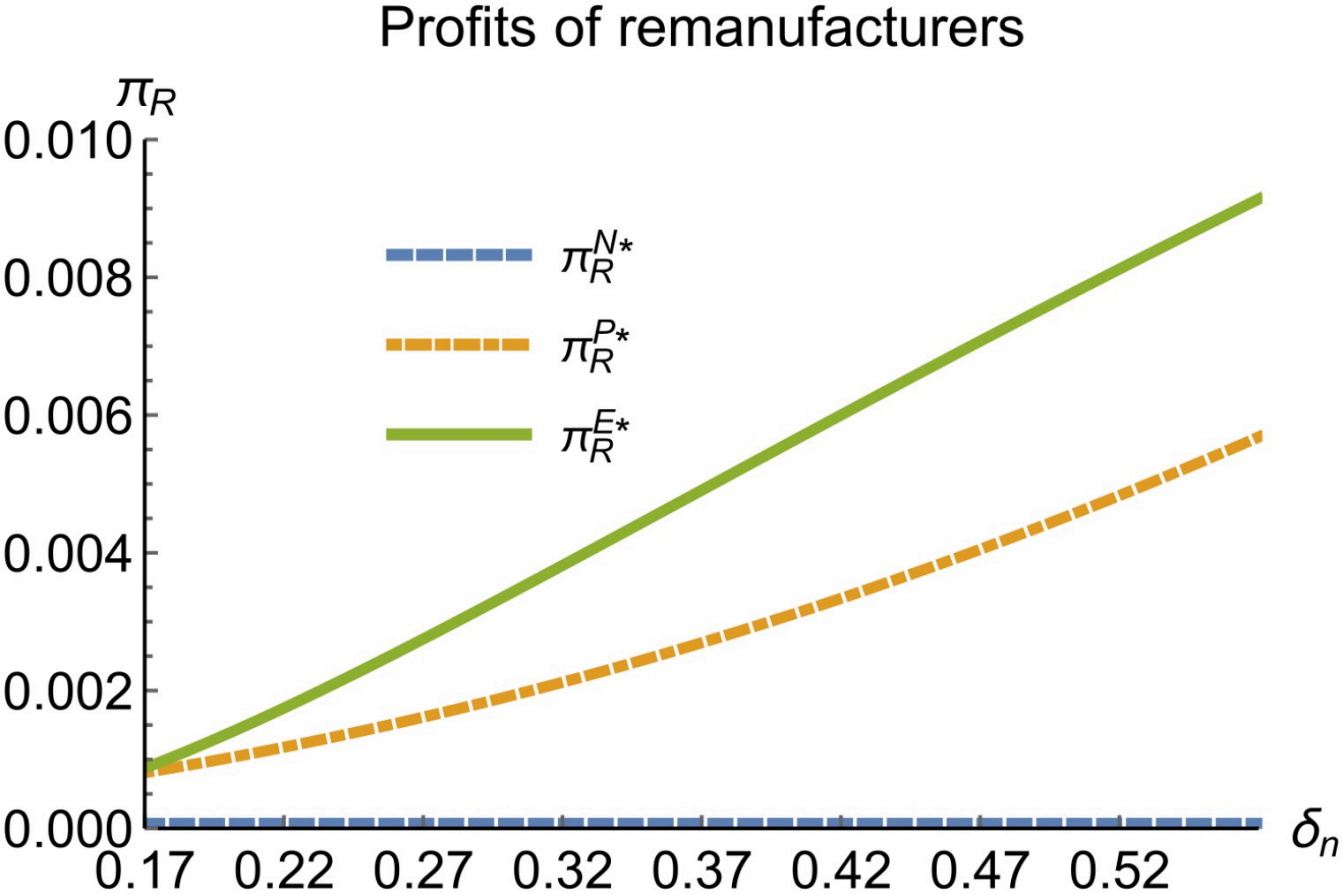
Profits of remanufacturers vary with *δ*_*n*_.

**Fig 20:**
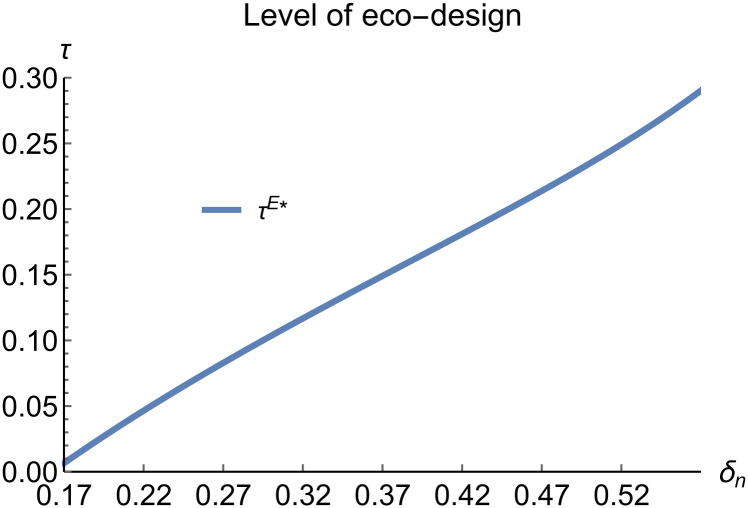
Level of eco-design vary with *δ*_*n*_.

When remanufacturing is unconstrained, we discuss the trends in equilibrium decisions as they change with the reward-penalty coefficient. The parameter assignments are as shown in Table 3. To ensure that 0 < *p*_*n*_ < 1, 0 < *p*_*r*_ < 1, 0 < *q*_*n*_,0 < *q*_*r*_, 0 < *π*_*M*_, 0 < *π*_*R*_, 0 < γ* < 1, 0 < *τ* < 1, $2k\bar{\alpha }-{\delta_{n}}^{2}>0$, we set *δ*_*n*_ to (0.17, 0.57). The simulation results are presented in Figs 14–20. The trend of equilibrium decision changes in the P mode is consistent with Proposition 3 (i).

**Table 3:** Parameter assignment.

parameter	*m*_*p*_	*α*	*c*_*n*_	*s*_*r*_	*k*	*δ*_*r*_	*m*_0_
assignment	0.1	0.46	0.43	0.15	0.31	0.25	0.55

In the E mode, as shown in Figs 14–20, with the increase in the reward-penalty coefficient, the price of new products first rises and then decreases, while the sales of new products initially decrease and then increase. In the early stages of increasing the reward-penalty coefficient, manufacturers raise the prices of new products, leading to a decline in sales as prices rise. As the reward-penalty coefficient continue to increase, manufacturers begin to lower the prices of new products. Once the price of new products decreases to a certain level, sales shift from declining to increasing. Manufacturer profits decline. With the increase in reward-penalty coefficient, the prices of remanufactured products decrease, sales increase, and remanufacturer profits rise. A higher reward-penalty coefficient is disadvantageous for manufacturers but beneficial for remanufacturers. A higher reward-penalty coefficient is favorable for consumers purchasing remanufactured products, while it may not necessarily disadvantage consumers buying new products. The level of eco-design increases with the rise in reward-penalty coefficient, and a higher coefficient is conducive to improving eco-design levels.

**Fig 21:**
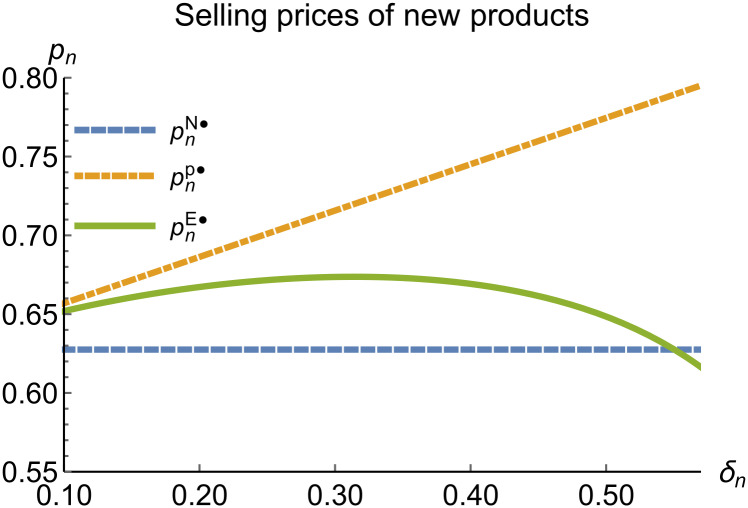
Selling prices of new products vary with *δ*_*n*_.

**Fig 22:**
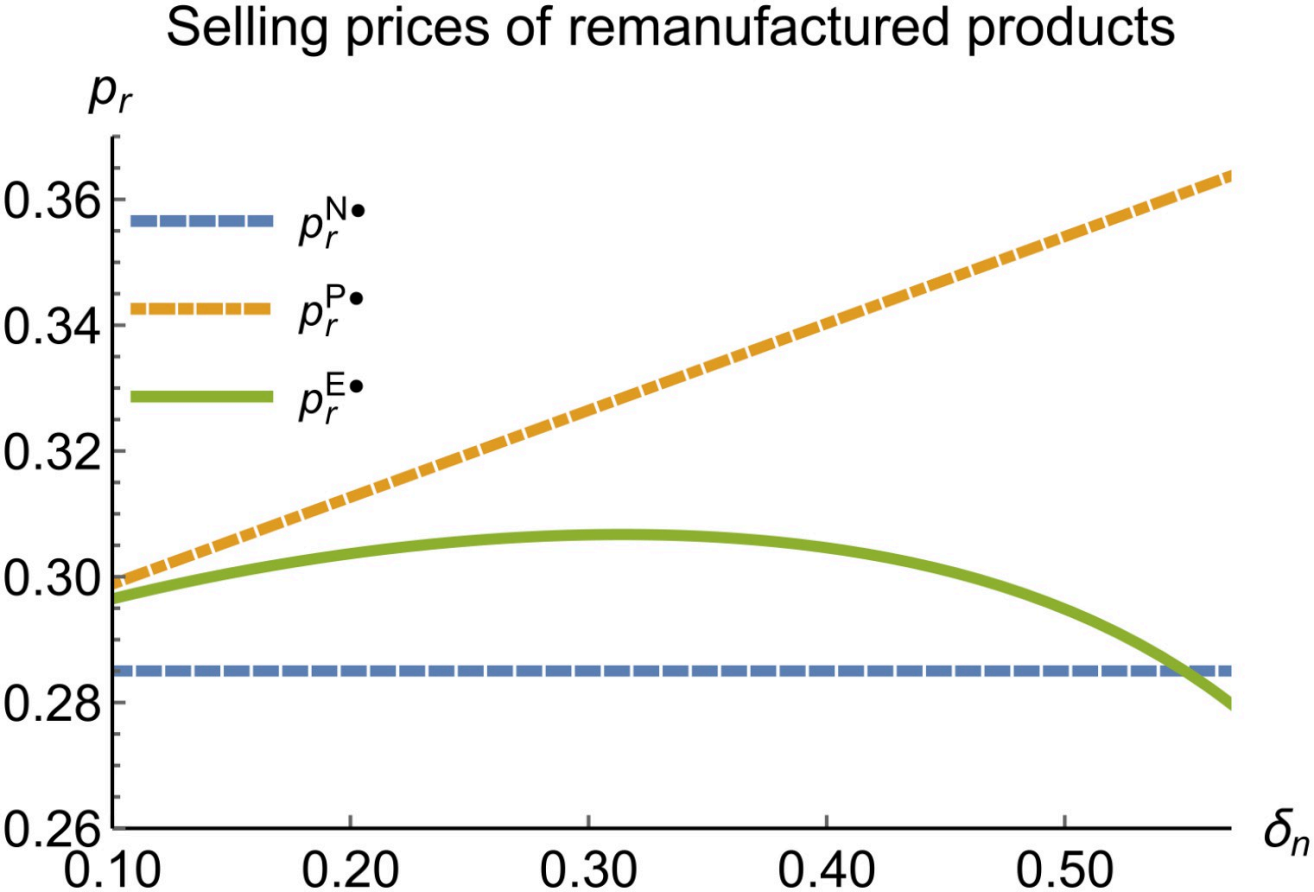
Selling prices of remanufactured products vary with *δ*_*n*_.

**Fig 23:**
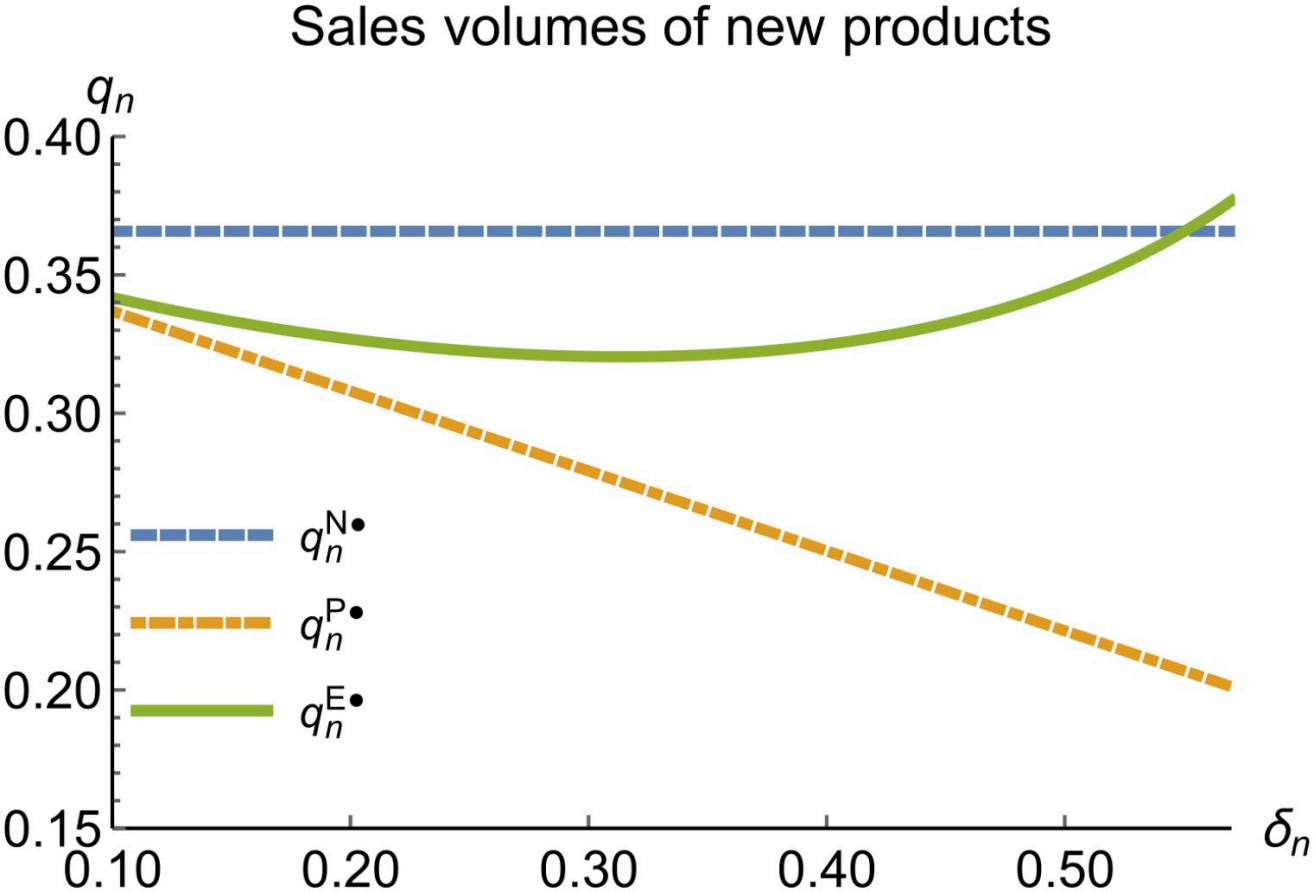
Sales volumes of new products vary with *δ*_*n*_.

**Fig 24:**
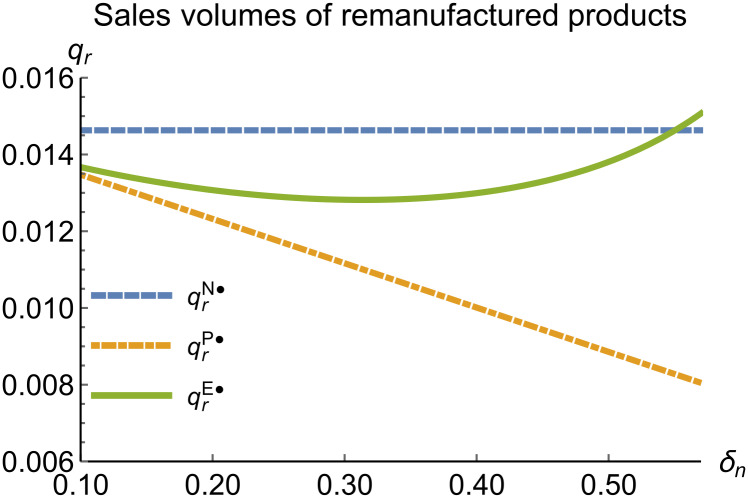
Sales volumes of remanufactured products vary with *δ*_*n*_.

**Fig 25:**
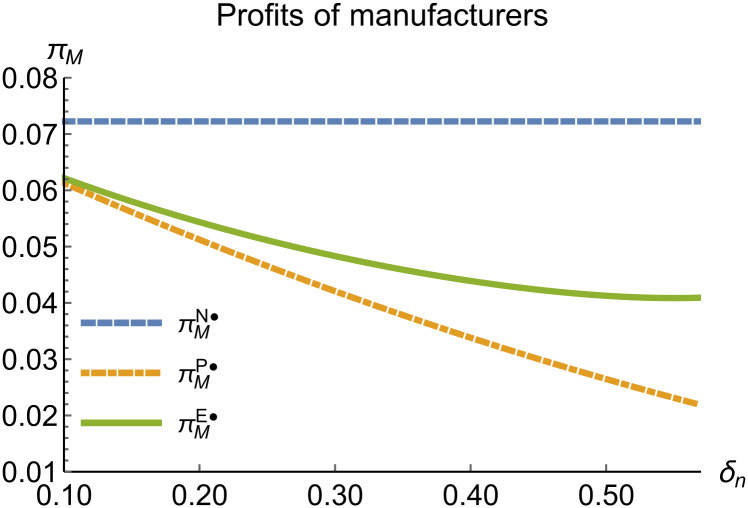
Profits of manufacturers vary with *δ*_*n*_.

**Fig 26:**
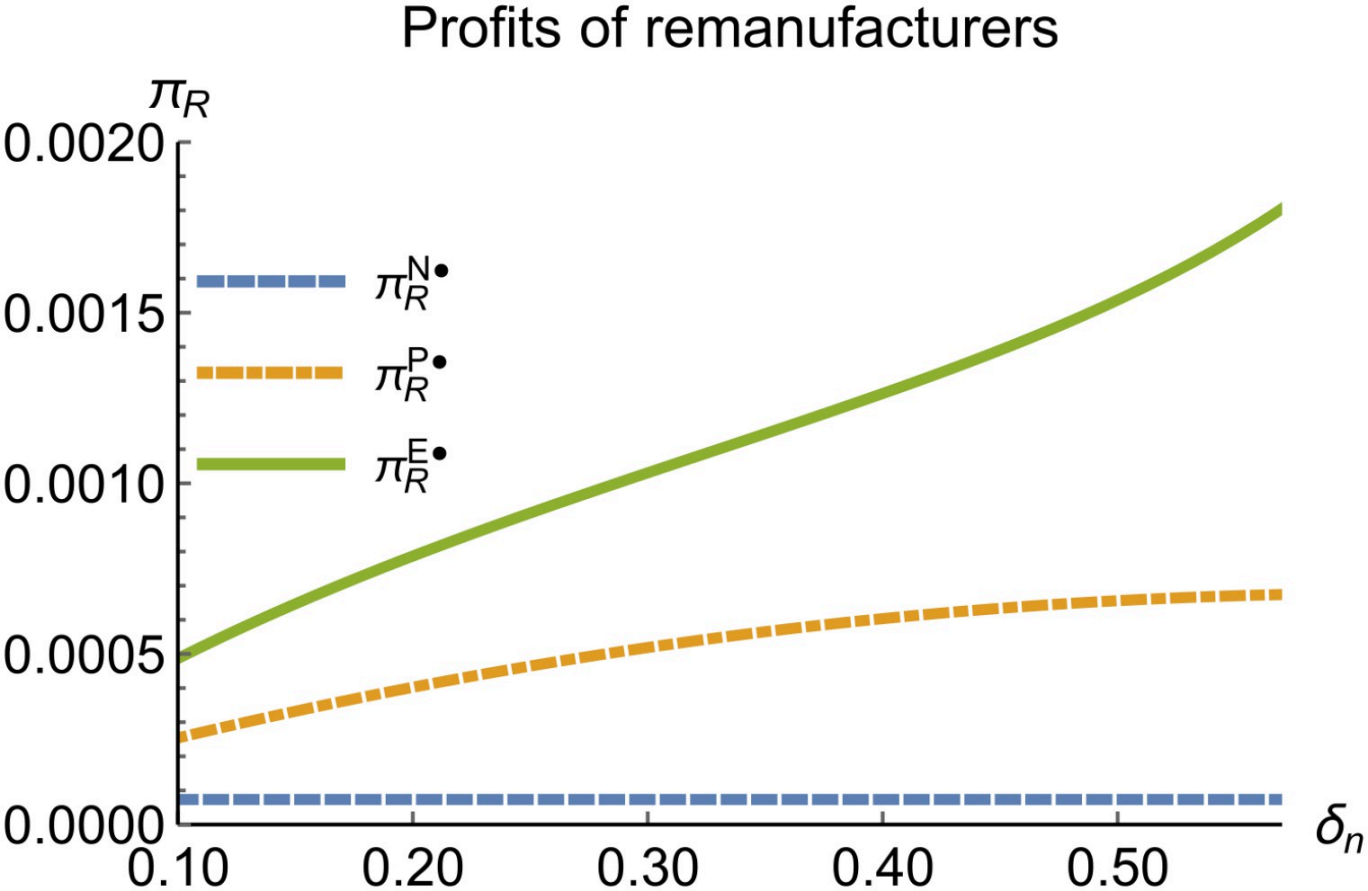
Profits of remanufacturers vary with *δ*_*n*_.

**Fig 27:**
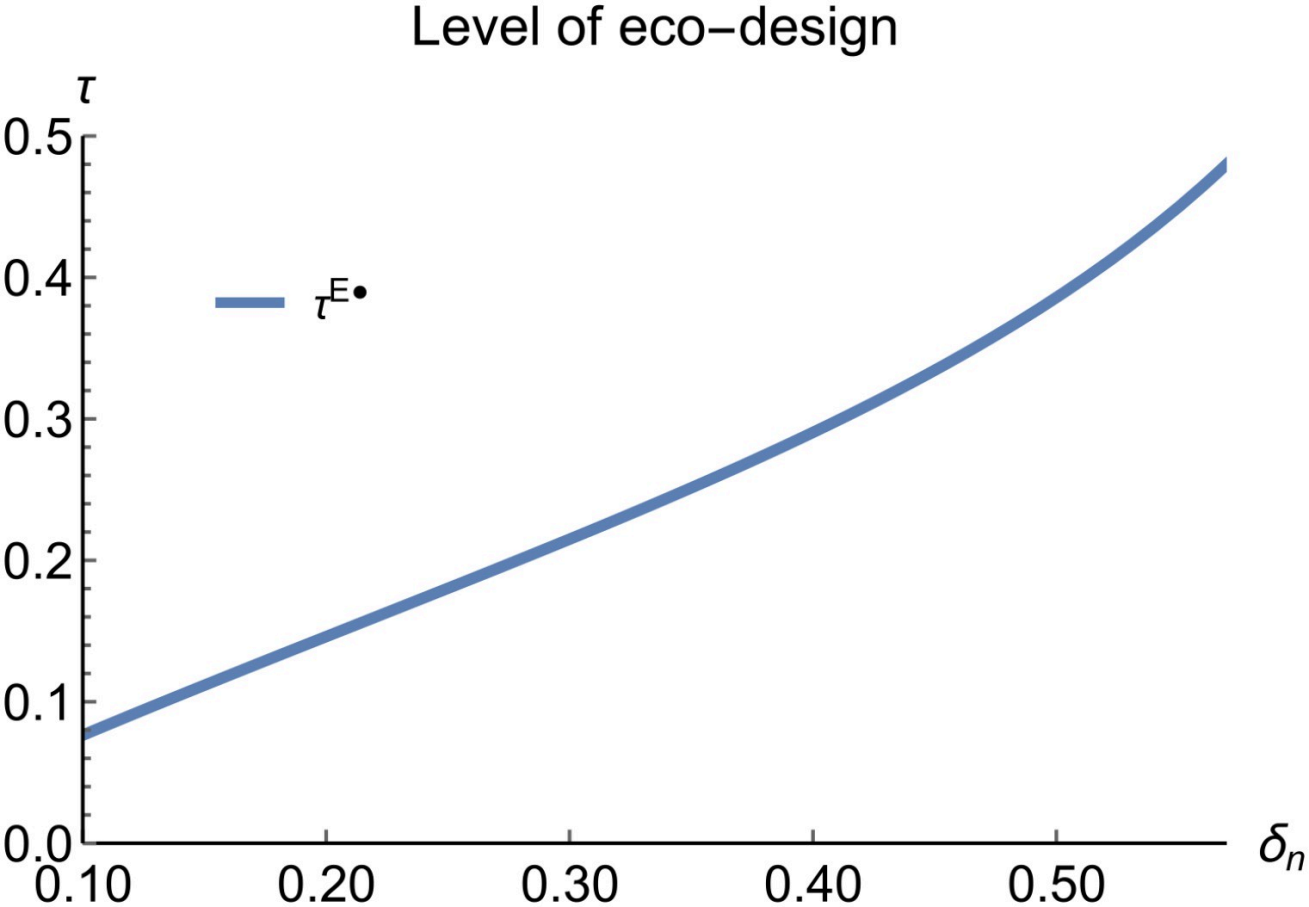
Level of eco-design vary with *δ*_*n*_.

When remanufacturing is constrained, we discuss the trends in equilibrium decisions as they change with the reward-penalty coefficient. Let *γ* = 0.04, with the remaining parameters assigned as shown in Table 3. To ensure that 0 < *p*_*n*_ < 1,0 < *p*_*r*_ < 1, 0 < *q*_*n*_,0 < *q*_*r*_,0 < *π*_*M*_, 0 < *π*_*R*_, *γ* < γ*, 0 < *τ* < 1 we set *δ*_*n*_ to (0.10, 0.57). The simulation results are presented in Figs 21–27. The trend of equilibrium decision changes in the P mode is consistent with Proposition 3 (ii).

As shown in Figs 21–27, in mode E, as the reward-penalty coefficient increases, the level of ecological design rises. The price of new products first increases and then decreases, while sales volume first declines and then increases. Remanufacturers, considering the limitations of remanufacturing, adopt a strategy where remanufactured product prices follow the changes of new product prices, first increasing and then decreasing. The sales volume of remanufactured products first declines and then increases. Manufacturer profits decrease, while remanufacturer profits increase.

Regardless of whether remanufacturing is restricted, a higher reward-penalty coefficient is beneficial for improving the level of ecological design, increasing remanufacturer profits, while manufacturer profits decrease. As the reward-penalty coefficient rises, the price of new products first increases and then decreases. The increase in the reward-penalty coefficient does not necessarily disadvantage consumers purchasing new products; once the coefficient reaches a certain level, it can actually benefit these consumers.

### 5.3 Comparison of equilibrium decisions

When remanufacturing is unrestricted, the comparison results of the equilibrium decisions between mode N and mode P, as shown in Figs 4–9 and 14–19, are consistent with Proposition 4 (i). The comparison results of the equilibrium decisions between mode P and mode E are consistent with Proposition 5 (i). From Figs 7 and 17, as well as Figs 9 and 19, it can be seen that the comparison results of the equilibrium decisions between mode N and mode E are consistent with Proposition 6 (i). From Figs 4–6, 8, 14–16 and 18, we conclude that that whether $p_{n}^{E*}-p_{n}^{N*}$, $p_{r}^{E*}-p_{r}^{N*}$, $q_{n}^{E*}-q_{n}^{N*}$, $\pi_{M}^{E*}-\pi_{M}^{N*}$ is greater than zero depends on the values of *δ*_*n*_ and *m*_0_.

When remanufacturing is restricted, the comparison results of the equilibrium decisions between mode N and mode P, as shown in Figs 11–13 and 21–26, are consistent with Proposition 4 (ii). The comparison results of the equilibrium decisions between mode P and mode E are consistent with Proposition 5 (ii). From Figs 13 and 26, it can be seen that the comparison results of the equilibrium decisions between mode N and mode E are consistent with Proposition 6 (ii). From Figs 11 and 21–25, we conclude that that whether $p_{n}^{E*}-p_{n}^{N*}$, $p_{r}^{E*}-p_{r}^{N*}$, $q_{n}^{E*}-q_{n}^{N*}$, $\pi_{M}^{E*}-\pi_{M}^{N*}$ is greater than zero depends on the values of *δ*_*n*_ and *m*_0_.

## 6 Conclusions and implications

### 6.1 Conclusions

We construct a closed-loop supply chain system consisting of a manufacturer and a remanufacturer. The manufacturer produces new products, and the remanufacturer recycles end-of-life products and produces remanufactured products. New and remanufactured products are priced differently. Governments set targets for the recycling rate of new products, imposing penalties on products that fall below the target and providing rewards for those that exceed it. The recycling rate target are set above the current levels of recycling achieved by the enterprises. Manufacturers can either choose not to take improvement measures and pay fines, or adopt ecological design to enhance product recycling rate. We studies the equilibrium decisions in three scenarios: when governments do not intervene, when governments set recycling targets but manufacturers do not take improvement measures, and when manufacturers adopt ecological design after governments establish recycling targets. The analysis considers both restricted and unrestricted remanufacturing situations, comparing the equilibrium decisions across the three scenarios, as well as examining the effects of recycling rate target and reward-penalty coefficient on these decisions. We draw the following conclusions:

(1) After government sets the recycling rate target, if manufacturers do not take improvement measures and accept economic penalties, the prices of new and remanufactured products will increase. Government’s penalty costs will be passed on to consumers, which is detrimental to consumers. The manufacturer’s profit will decrease, while the remanufacturer’s profit will increase.
(2) After manufacturers adopt eco-design, the prices of both new and remanufactured products decrease, and their sales increase. Eco-design benefits consumers and helps expand the market share of both new and remanufactured products. The profits of both manufacturers and remanufacturers increase. Therefore, when government sets a recycling rate target, manufacturers would be well-advised to adopt eco-design to improve the recycling rate of their products, regardless of whether remanufacturing is restricted.
(3) After manufacturers adopt ecological design, as the recycling rate target increase, the level of ecological design for new products decreases, while the prices of both new and remanufactured products rise, leading to a decrease in manufacturer profits. When remanufacturing is unrestricted, the sales volume of remanufactured products increases, and remanufacturer profits rise. When remanufacturing is restricted, the sales volume of remanufactured products decreases, and remanufacturer profits first increase and then decline. Therefore, setting lower recycling rate target is beneficial for improving the level of ecological design and also advantageous for consumers. It is recommended that governments initially set lower recycling rate targets and then gradually increase them.
(4) After manufacturers adopt ecological design, as the reward-penalty coefficient increases, the level of ecological design rises, and the price of new products first increases and then decreases. Manufacturer profits decrease while remanufacturer profits increase. When remanufacturing is unrestricted, an increase in reward-penalty coefficient leads to a decrease in the prices of remanufactured products. When remanufacturing is restricted, the prices of remanufactured products first increase and then decrease. Therefore, setting a higher reward-penalty coefficient is beneficial for improving the level of ecological design and does not necessarily disadvantage consumers; once the coefficient reaches a certain level, it can actually benefit consumers. Governments would be well-advised to establish a relatively high reward-penalty coefficient.

### 6.2 Implications

When government sets recycling rate target, the adoption of eco-design by manufacturers benefits consumers and helps increase the profits of both manufacturers and remanufacturers. Based on the research conclusions, the following management recommendations are proposed.

(1) Government would be well-advised to encourage and guide companies to adopt eco-design by setting recycling rate target. The purpose of these targets is to encourage companies to improve the recycling rate of their products from their initial design stage, rather than merely requiring them to pay fines as a form of taxation. Along with setting recycling rate target, government would be well-advised to also implement measures such as public education and industry guidance to encourage companies to adopt eco-design.
(2) Manufacturers would be well-advised to adopt improvement measures such as ecological design to enhance the product’s recycling rate from the source. Following the European Union’s establishment of recycling rate target for automotive and electronic products, China has also set corresponding recycling rate target. In the future, more industries will face issues related to recycling rate target. Companies would be well-advised to take proactive measures, such as adopting ecological design and other improvement strategies, to enhance the recycling rate of their products.
(3) Government would be well-advised to set lower initial recycling rate target and higher reward-penalty coefficient. Government would be well-advised to establish recycling rate target based on current recycling levels, starting with lower targets and gradually increasing them in phases to incentivize innovation and progress. A higher reward-penalty coefficient would be well-advised to be implemented to severely penalize companies that do not meet the targets and reward those that do.

## Supporting information

S1 File Proof of proposition. This file contains the detailed proofs of Proposition 1(i), Proposition 1(ii), Proposition 2(i), Proposition 2(ii), Proposition 3(i), Proposition 3(ii), Proposition 4(i), Proposition 4(ii), Proposition 5(i), and Proposition 5(ii).
(PDF)
