# Supplementary material for: Impact of manufacturers’ eco-design decisions on the closed-loop supply chain under recycling rate regulations
Source: PLoS One. 2025 Feb 11;20(2):e0314511. doi: 10.1371/journal.pone.0314511 (PMC11813158; doi:10.1371/journal.pone.0314511)
Supplement: S1 File — This file contains the detailed proofs of Proposition 1(i), Proposition 1(ii), Proposition 2(i), Proposition 2(ii), Proposition 3(i), Proposition 3(ii), Proposition 4(i), Proposition 4(ii), Proposition 5(i), and Proposition 5(ii). (PDF) [file pone.0314511.s001.pdf]

Supporting information

S1 Proof of Proposition 1(i) $\frac{\partial p_n^{P*}}{\partial m_o} = \frac{2\delta_n}{3+\bar{\alpha}} > 0,\ \frac{\partial p_r^{P*}}{\partial m_o} = \frac{\alpha\delta_n}{3+\bar{\alpha}} > 0,$
$\frac{\partial q_n^{P*}}{\partial m_o} = -\frac{(1+\bar{\alpha})\delta_n}{(3+\bar{\alpha})\bar{\alpha}} < 0,\ \frac{\partial q_r^{P*}}{\partial m_o} = \frac{\delta_n}{(3+\bar{\alpha})\bar{\alpha}} > 0,\ \frac{\partial \pi_M^{P*}}{\partial m_o} = -\frac{2(1+\bar{\alpha})\delta_n((2-c_n)\bar{\alpha}-s_r-m(1+\bar{\alpha})\delta_n)}{(3+\bar{\alpha})^2\bar{\alpha}} < 0,$
$\frac{\partial \pi_R^{P*}}{\partial m_o} = \frac{2\delta_n(s_r+\bar{\alpha}(\alpha-2c_n+s_r)+m_o\alpha\delta_n)}{(3+\bar{\alpha})^2\bar{\alpha}} > 0,\ \frac{\partial \gamma^{P*}}{\partial m_o} = \frac{\bar{\alpha}(3+\bar{\alpha})(\alpha-c_n+s_r)\delta_n}{\alpha(2\bar{\alpha}-\bar{\alpha}c_n-s_r-m(1-\bar{\alpha})\delta_n)^2} > 0,$
$\frac{\partial p_n^{P*}}{\partial m_o} - \frac{\partial p_r^{P*}}{\partial m_o} = \frac{(1+\bar{\alpha})\delta_n}{3+\bar{\alpha}} > 0.$

S1 Proof of Proposition 1(ii) $\frac{\partial p_n^{P\bullet}}{\partial m_o} = \frac{(1+\alpha\gamma)\delta_n}{2-\alpha\bar{\gamma}} > 0,$
$\frac{\partial p_r^{P\bullet}}{\partial m_o} = \frac{(1+\alpha\gamma)\delta_n}{2-\alpha\bar{\gamma}} > 0, \frac{\partial q_n^{P\bullet}}{\partial m_o} = \frac{-\delta_n}{2-\alpha\bar{\gamma}} < 0, \frac{\partial q_r^{P\bullet}}{\partial m_o} = \frac{-\gamma\delta_n}{2-\alpha\bar{\gamma}} < 0,$
$\frac{\partial \pi_M^{P\bullet}}{\partial m_o} = -\frac{2\bar{\alpha}\delta_n(\bar{c}_n-m\delta_n)}{(2-\alpha\bar{\gamma})^2} < 0, \frac{\partial^2 \pi_R^{P\bullet}}{\partial {m_o}^2} = -\frac{2\alpha\gamma(1+\gamma)\delta_n^2}{(2-\alpha\bar{\gamma})^2} < 0.$ Proof complete.

S2 Proof of Proposition 2(i) $\frac{\partial \tau^{E*}}{\partial m_o} = -\frac{2(1+\bar{\alpha})\delta_n((1+\bar{\alpha})\delta_n-\delta_r)}{k\bar{\alpha}(3+\bar{\alpha})^2-2((1+\bar{\alpha})\delta_n-\delta_r)^2} < 0,$
$\frac{\partial p_n^{E*}}{\partial m_o} = \frac{2\delta_n\left(k\bar{\alpha}(3+\bar{\alpha})+(1+\bar{\alpha})\delta_n\delta_r-\delta_r^2\right)}{k\bar{\alpha}(3+\bar{\alpha})^2-2((1+\bar{\alpha})\delta_n-\delta_r)^2} > 0,\ \frac{\partial p_r^{E*}}{\partial m_o} = \frac{\delta_n\left(k\alpha\bar{\alpha}(3+\bar{\alpha})+2(1+\bar{\alpha})\delta_n\delta_r-2\delta_r^2\right)}{k\bar{\alpha}(3+\bar{\alpha})^2-2((1+\bar{\alpha})\delta_n-\delta_r)^2} > 0,$
$\frac{\partial q_n^{E*}}{\partial m_o} = -\frac{(1+\bar{\alpha})\delta_n}{\bar{\alpha}(3+\bar{\alpha})} < 0,\ \frac{\partial q_r^{E*}}{\partial m_o} = \frac{\delta_n}{3\bar{\alpha}+\bar{\alpha}^2} > 0.$ From $\gamma* = \frac{q_r^{E*}}{q_n^{E*}} < 1$, the maximum value of m can be determined, substituting it into $\frac{\partial \pi_M^{E*}}{\partial m_o}$, we obtain
$\frac{\partial \pi_M^{E*}}{\partial m_o} < \frac{-2k(1+\bar{\alpha})(\alpha-c_n+s_r)\delta_n}{k\alpha(3+\bar{\alpha})-2(1+\bar{\alpha})\delta_n\delta_r+2\delta_r^2} < 0.$ Proof complete.

Similarly, $\frac{\partial \pi_R^{E*}}{\partial m_o} = \frac{2\delta_n(s_r+\alpha(m-\tau)\delta_n+\tau\delta_r+\bar{\alpha}(\alpha-2c_n+s_r+\tau\delta_r))}{\bar{\alpha}(3+\bar{\alpha})^2} > 0,$
$\frac{\partial \gamma^{E*}}{\partial m_o} = \frac{(\alpha-c_n+s_r)\delta_n\left(k\bar{\alpha}(3+\bar{\alpha})^2-2((1+\bar{\alpha})\delta_n-\delta_r)^2\right)}{k\alpha(3+\bar{\alpha})(2\bar{\alpha}-\bar{\alpha}c_n-s_r-m(1+\bar{\alpha})\delta_n)^2} > 0,$
$\frac{\partial p_n^{E*}}{\partial m_o} - \frac{\partial p_r^{E*}}{\partial m_o} = \frac{k\delta_n\bar{\alpha}(3+\bar{\alpha})}{k\bar{\alpha}(3+\bar{\alpha})^2-2((1+\bar{\alpha})\delta_n-\delta_r)^2} > 0.$

S2 Proof of Proposition 2(ii)
$\frac{\partial \tau^{E\bullet}}{\partial m_o} = -\frac{2\bar{\alpha}\delta_n^2}{k(2-\alpha\bar{\gamma})^2-2\bar{\alpha}\delta_n^2} < 0, \frac{\partial p_n^{E\bullet}}{\partial m_o} = \frac{k(1+\alpha\gamma)(2-\alpha\bar{\gamma})\delta_n}{k(2-\alpha\bar{\gamma})^2-2\bar{\alpha}\delta_n^2} > 0,$
$\frac{\partial p_r^{E\bullet}}{\partial m_o} = \frac{k\alpha(1+\gamma)(2-\alpha\bar{\gamma})\delta_n}{k(2-\alpha\bar{\gamma})^2-2\bar{\alpha}\delta_n^2} > 0, \frac{\partial q_n^{E\bullet}}{\partial m_o} = \frac{-k(2-\alpha\bar{\gamma})\delta_n}{k(2-\alpha\bar{\gamma})^2-2\bar{\alpha}\delta_n^2} < 0,$
$\frac{\partial q_r^{E\bullet}}{\partial m_o} = \frac{-k\gamma(2-\alpha\bar{\gamma})\delta_n}{k(2-\alpha\bar{\gamma})^2-2\bar{\alpha}\delta_n^2} < 0, \frac{\partial \pi_M^{E\bullet}}{\partial m_o} = -\frac{2k\bar{\alpha}\delta_n(\bar{c}_n-m\delta_n)}{k(2-\alpha\bar{\gamma})^2-2\bar{\alpha}\delta_n^2} < 0,$
$\frac{\partial^2 \pi_R^{E\bullet}}{\partial {m_o}^2} = -\frac{2k\gamma(2-\alpha\bar{\gamma})\delta_n^2(k\alpha(1+\gamma)(2-\alpha\bar{\gamma})-2\bar{\alpha}\delta_n\delta_r)}{\left(k(2-\alpha\bar{\gamma})^2-2\bar{\alpha}\delta_n^2\right)^2} < 0$ can be proved. Proof complete.

S3 Proof of Proposition 3(i) $\frac{\partial p_n^{P*}}{\partial \delta_n} = \frac{2m}{3+\bar{\alpha}} > 0, \frac{\partial p_r^{P*}}{\partial \delta_n} = \frac{\alpha m}{3+\bar{\alpha}} > 0,$
$\frac{\partial q_n^{P*}}{\partial \delta_n} = -\frac{m(1+\bar{\alpha})}{(3+\bar{\alpha})\bar{\alpha}} < 0,\ \frac{\partial q_r^{P*}}{\partial \delta_n} = \frac{m}{(3+\bar{\alpha})\bar{\alpha}} > 0,\ \frac{\partial \pi_M^{P*}}{\partial \delta_n} = -\frac{2m(1+\bar{\alpha})((2-c_n)\bar{\alpha}-s_r-m(1+\bar{\alpha})\delta_n)}{(3+\bar{\alpha})^2\bar{\alpha}} < 0,$
$\frac{\partial \pi_R^{P*}}{\partial \delta_n} = \frac{2m(s_r+\bar{\alpha}(\alpha-2c_n+s_r)+m\alpha\delta_n)}{(3+\bar{\alpha})^2\bar{\alpha}} > 0,$
$\frac{\partial \gamma^{P*}}{\partial \delta_n} = \frac{m\bar{\alpha}(3+\bar{\alpha})(\alpha-c_n+s_r)}{\alpha(2\bar{\alpha}-\bar{\alpha}c_n-s_r-m(1+\bar{\alpha})\delta_n)^2} > 0, \frac{\partial p_n^{P*}}{\partial \delta_n} - \frac{\partial p_r^{P*}}{\partial \delta_n} = \frac{(1+\bar{\alpha})m}{3+\bar{\alpha}} > 0.$ Proof complete.

S3 Proof of Proposition 3(ii) $\frac{\partial p_n^{P\bullet}}{\partial \delta_n} = \frac{m(1+\alpha\gamma)}{2-\alpha\bar{\gamma}} > 0$, $\frac{\partial p_r^{P\bullet}}{\partial \delta_n} = \frac{m\alpha(1+\gamma)}{2-\alpha\bar{\gamma}} > 0, \frac{\partial q_n^{P\bullet}}{\partial \delta_n} = \frac{-m}{2-\alpha\bar{\gamma}} < 0, \frac{\partial q_r^{P\bullet}}{\partial \delta_n} = \frac{-m\gamma}{2-\alpha\bar{\gamma}} < 0$, $\frac{\partial \pi_M^{P\bullet}}{\partial \delta_n} = -\frac{2m\bar{\alpha}(\bar{c}_n - m\delta_n)}{(2-\alpha\bar{\gamma})^2} < 0, \frac{\partial^2 \pi_R^{P\bullet}}{\partial {\delta_n}^2} = -\frac{2m^2\alpha\gamma(1+\gamma)}{(2-\alpha\bar{\gamma})^2} < 0$. Proof complete.

S4 Proof of Proposition 4(i) $p_n^{P*} - p_n^{N*} = \frac{2m\delta_n}{3+\bar{\alpha}} > 0$, $p_r^{P*} - p_r^{N*} = \frac{m\alpha\delta_n}{3+\bar{\alpha}} > 0$, $q_n^{N*} - q_n^{P*} = \frac{m(1+\bar{\alpha})\delta_n}{(3+\bar{\alpha})\bar{\alpha}} > 0$, $q_r^{P*} - q_r^{N*} = \frac{m\delta_n}{4-5\alpha+\alpha^2} > 0$, $\pi_M^{N*} - \pi_M^{P*} = \frac{m(1+\bar{\alpha})\delta_n(2(\bar{\alpha}(2-c_n)-s_r)-m(1+\bar{\alpha})\delta_n)}{\bar{\alpha}(3+\bar{\alpha})^2}$, $2\left(\bar{\alpha}\left(2-c_n\right)-s_r\right) - m\left(1+\bar{\alpha}\right)\delta_n$ is a decreasing function of m. From $m < 1$, we obtain $\pi_M^{N*} - \pi_M^{P*} > \frac{(1+\bar{\alpha})\delta_n(2(\bar{\alpha}(2-c_n)-s_r)-(1+\bar{\alpha})\delta_n)}{\bar{\alpha}(3+\bar{\alpha})^2} > 0$. Similarly, $\pi_R^{P*} - \pi_R^{N*} > 0$ can be proved. Proof complete.

S4 Proof of Proposition 4(ii) $p_n^{P\bullet} - p_n^{N\bullet} = \frac{(m+m\alpha\gamma)\delta_n}{2-\alpha\bar{\gamma}} > 0$, $p_r^{P\bullet} - p_r^{N\bullet} = \frac{m\alpha(1+\gamma)\delta_n}{2-\alpha\bar{\gamma}} > 0$, $q_n^{P\bullet} - q_n^{N\bullet} = -\frac{m\delta_n}{2-\alpha\bar{\gamma}} < 0$, $q_r^{P\bullet} - q_r^{N\bullet} = -\frac{m\gamma\delta_n}{2-\alpha\bar{\gamma}} < 0$, $\pi_M^{P\bullet} - \pi_M^{N\bullet} = -\frac{m\bar{\alpha}\delta_n(2\bar{c}_n - m\delta_n)}{(2-\alpha\bar{\gamma})^2} < 0$, $\pi_R^{P\bullet} - \pi_R^{N\bullet} = \frac{m\gamma\delta_n((2-\alpha(3+\gamma))c_n-(2-\alpha\bar{\gamma})s_r+\alpha(\alpha+\gamma(1+\bar{\alpha})-m(1+\gamma)\delta_n))}{(2-\alpha\bar{\gamma})^2} > 0$. Proof complete.

S5 Proof of Proposition 5(i) $p_n^{P*} - p_n^{E*} > \frac{2\bar{\alpha}(2\delta_n+\delta_r)((1+\bar{\alpha})\delta_n-\delta_r)}{k(3+\bar{\alpha})^2\bar{\alpha}-2((1+\bar{\alpha})\delta_n-\delta_r)^2} > 0$, $p_r^{P*} - p_r^{E*} > \frac{2\bar{\alpha}((1+\bar{\alpha})\delta_n-\delta_r)(\alpha\delta_n+2\delta_r)}{k(3+\bar{\alpha})^2\bar{\alpha}-2((1+\bar{\alpha})\delta_n-\delta_r)^2} > 0$, $q_n^{E*} - q_n^{P*} > \frac{2((1+\bar{\alpha})\delta_n-\delta_r)^2}{k(3+\bar{\alpha})^2\bar{\alpha}-2((1+\bar{\alpha})\delta_n-\delta_r)^2} > 0$, $q_r^{E*} - q_r^{P*} > \frac{2((1+\bar{\alpha})\delta_n-\delta_r)((1+\bar{\alpha})\delta_r-\alpha\delta_n)}{\alpha\left(k(3+\bar{\alpha})^2\bar{\alpha}-2((1+\bar{\alpha})\delta_n-\delta_r)^2\right)} > 0$, $\pi_M^{E*} - \pi_M^{P*} > \frac{2\bar{\alpha}((1+\bar{\alpha})\delta_n-\delta_r)^2}{k(3+\bar{\alpha})^2\bar{\alpha}-2((1+\bar{\alpha})\delta_n-\delta_r)^2} > 0$, $\pi_R^{E*} - \pi_R^{P*} > 0$.

S5 Proof of Proposition 5(ii) $p_n^{P\bullet} - p_n^{E\bullet} = \frac{2(1+\alpha\gamma)\bar{\alpha}\delta_n^2(\bar{c}_n-m\delta_n)}{k(2-\alpha\bar{\gamma})^3-2\bar{\alpha}(2-\alpha\bar{\gamma})\delta_n^2} > 0$, $p_r^{P\bullet} - p_r^{E\bullet} = \frac{2\alpha(1+\gamma)\bar{\alpha}\delta_n^2(\bar{c}_n-m\delta_n)}{k(2-\alpha\bar{\gamma})^3-2\bar{\alpha}(2-\alpha\bar{\gamma})\delta_n^2} > 0$, $q_n^{E\bullet} - q_n^{P\bullet} = \frac{2\bar{\alpha}\delta_n^2(\bar{c}_n-m\delta_n)}{k(2-\alpha\bar{\gamma})^3-2\bar{\alpha}(2-\alpha\bar{\gamma})\delta_n^2} > 0$, $q_r^{E\bullet} - q_r^{P\bullet} = \frac{2\gamma\bar{\alpha}\delta_n^2(\bar{c}_n-m\delta_n)}{k(2-\alpha\bar{\gamma})^3-2\bar{\alpha}(2-\alpha\bar{\gamma})\delta_n^2} > 0$, $\pi_M^{E\bullet} - \pi_M^{P\bullet} = \frac{2\bar{\alpha}^2\delta_n^2(\bar{c}_n-m\delta_n)^2}{k(2-\alpha\bar{\gamma})^4-2\bar{\alpha}(2-\alpha\bar{\gamma})^2\delta_n^2} > 0$, $\pi_R^{E\bullet} - \pi_R^{P\bullet} > 0$. Proof complete.
